# Morphological diversity and molecular phylogeny of five *Paramecium bursaria* (Alveolata, Ciliophora, Oligohymenophorea) syngens and the identification of their green algal endosymbionts

**DOI:** 10.1038/s41598-022-22284-z

**Published:** 2022-10-27

**Authors:** Christian Spanner, Tatyana Darienko, Sabine Filker, Bettina Sonntag, Thomas Pröschold

**Affiliations:** 1 Research Department for Limnology, Mondsee, University of Innsbruck, Mondsee, Austria; 2 Institute of Microbiology and Genetics, Department of Applied Bioinformatics, University of Göttingen, Göttingen, Germany; 3 Molecular Ecology Group, Technische Universität Kaiserslautern, Kaiserslautern, Germany

**Keywords:** Cell biology, Ecology, Evolution, Limnology

## Abstract

*Paramecium bursaria* is a mixotrophic ciliate species, which is common in stagnant and slow-flowing, nutrient-rich waters. It is usually found living in symbiosis with *zoochlorellae* (green algae) of the genera *Chlorella* or *Micractinium*. We investigated *P. bursaria* isolates from around the world, some of which have already been extensively studied in various laboratories, but whose morphological and genetic identity has not yet been completely clarified. Phylogenetic analyses of the SSU and ITS rDNA sequences revealed five highly supported lineages, which corresponded to the syngen and most likely to the biological species assignment. These syngens R1–R5 could also be distinguished by unique synapomorphies in the secondary structures of the SSU and the ITS. Considering these synapomorphies, we could clearly assign the existing GenBank entries of *P. bursaria* to specific syngens. In addition, we discovered synapomorphies at amino acids of the COI gene for the identification of the syngens. Using the metadata of these entries, most syngens showed a worldwide distribution, however, the syngens R1 and R5 were only found in Europe. From morphology, the syngens did not show any significant deviations. The investigated strains had either *Chlorella variabilis*, *Chlorella vulgaris* or *Micractinium conductrix* as endosymbionts.

## Introduction

*Paramecium bursaria* has been studied since decades because of its easiness to be kept and experimentally manipulated under manifold cultivation conditions. Some major aspects on this model ciliate were investigated in detail: (i) *P. bursaria* lives in symbiosis with coccoid green algae belonging to the genera *Chlorella* and *Micractinium* (Pröschold et al.^1^ and references therein). Advantages of this close relationship include nutritional aspects as the algae provide photosynthetic products and photoprotection to the ciliate^2,3^. Accordingly, different aspects such as the process of cell–cell recognition and the symbiont-specificity are of great interest (Fujishima^4^ and articles therein). (ii) Complex mating systems in *P. bursaria* were discovered in mating experiments during (sexual) conjugation processes. So far, six genetic varieties were originally detected by Sonneborn^5^ and later designated as syngens 1 to 6, which were considered as biological species^6–8^. Most syngen-types have four (syngens 1 and 3) or eight (syngens 2 and 4–6) mating types^9^. As the strains that Bomford^9^ used for his experiments were lost, Greczek-Stachura et al.^10^ established a new syngen system, i.e., R1-R5 in principle most likely corresponding to Bomford’s syngens and indicated by a “B” but in a different order (R1–B6, R2–B4, R3–B1, R4–B2, and R5—B3 according to Greczek-Stachura et al.^10^). Syngen 5 of Bomford^9^ was not included. However, the subdivision into syngens was accompanied by phylogenetic analyses of the ITS (internal transcribed spacer regions; partial SSU–ITS1–5.8S–ITS2–partial LSU region) rDNA, the mitochondrial COI (cytochrome oxidase I) and H4 histone genes^10^. The five syngens were recently described as cryptic species based on COI haplotypes and named accordingly as *Paramecium primabursaria*, *P*. *bibursaria*, *P*. *tribursaria*, *P*. *tetrabursaria* and, *P*. *pentabursaria*^11^. Unfortunately, these species were not validly described according to the International Code for Zoological Nomenclature (ICZN), which requires formal descriptions and deposition of holotype specimens to public museums.

Despite such detailed studies in respect to conjugation and endosymbiosis, the morphology and the phenotypic plasticity of *P. bursaria* has only been rarely investigated. Kreutz et al.^12^ compared the morphology and ultrastructure of one *P. bursaria* strain with another “green” *Paramecium*, i.e., *Paramecium chlorelligerum*. However, both species were investigated directly from field samples and the phenotypic plasticity was not studied from cultured material.

The aim of this study was the comparison of 48 *P. bursaria* strains using an integrative approach to answer the following questions: (i) How many phylogenetic lineages among the investigated strains can be revealed? (ii) Do they correspond to the known syngen affiliations? (iii) Does the morphology of the ciliate strains differ among the syngens? (iv) Do the different syngens show any biogeographic pattern? and, (v) Do all strains bear the same algal endosymbiont? We studied the strains both isolated from diverse geographical regions and acquired from culture collections. First, we sequenced the SSU and ITS rDNA sequences. Subsequently, from each phylogenetic clade, at least one strain was selected to study its morphology and phenotypic plasticity from living and silver-stained specimens. Finally, the green algal endosymbionts were identified both from morphology and a diagnostic PCR approach.

## Results

### Molecular Phylogeny of *Paramecium bursaria* and Identification of its Endosymbionts

**Figure 1:**
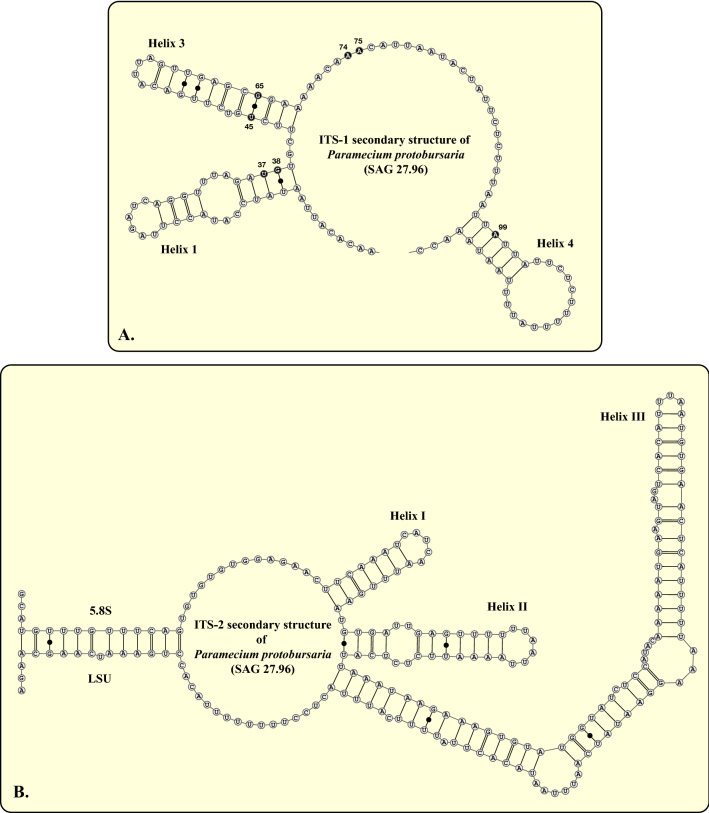
ITS‐1 (**A**) and ITS-2 (**B**) secondary structures of *Paramecium protobursaria*, SAG 27.96 (syngen R1).

**Figure 2:**
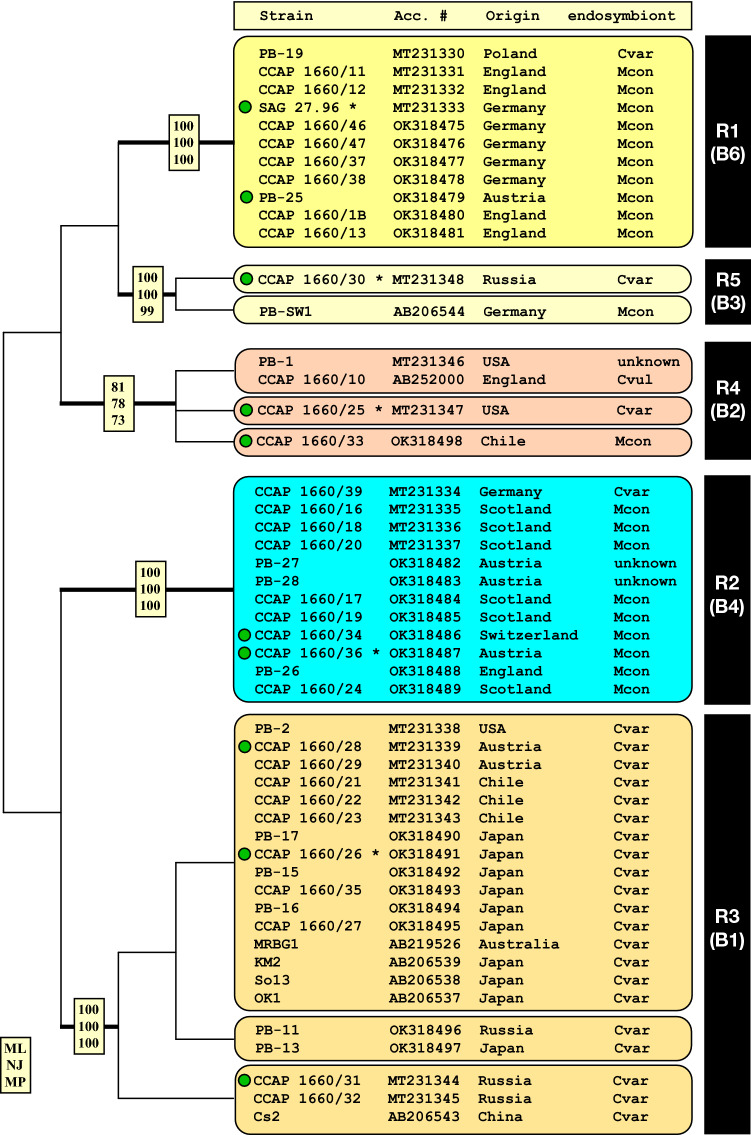
Molecular phylogeny of the *Paramecium bursaria* species complex based on SSU and ITS rDNA sequence comparisons. The phylogenetic tree shown was inferred using the maximum likelihood method based on the datasets (2197 aligned positions of 19 taxa) using the computer program PAUP 4.0a169. For the analyses, the best model was calculated by PAUP 4.0a169. The setting of the best model was given as follows: TVM + I (base frequencies: A 0.2983, C 0.1840, G 0.2271, T 0.2906; rate matrix A–C 2.6501, A–G 8.6851, A–U 5.3270, C–G 0.91732, C–U 8.6851, G–U 1.0000) with the proportion of invariable sites (I = 0.9544). The branches in bold are highly supported in all bootstrap analyses (bootstrap values > 50% calculated with PAUP using the maximum likelihood, neighbour—joining, and maximum parsimony). The clades are named after the syngens (color‐coded) proposed by Greczek‐Stachura et al.^10^ and Bomford^9^ in brackets. The accession numbers are given after the strain numbers. The endosymbiotic green algae identified are highlighted (Mcon—*Micractinium conductrix*, Cvar—*Chlorella variabilis* and Cvul—*Chlorella vulgaris*) after the origin of the *P. bursaria* strains. The reference strain of each syngen is marked with an asterisk. The strains used for morphological comparisons are marked with a green dot next to the strain number.

The SSU and ITS rDNA of the nuclear ribosomal operon were sequenced to infer the genetic variability of the investigated strains. The SSU and ITS rDNA sequences were aligned according to their secondary structure (examples are presented for the strain SAG 27.96; Fig. 1 and Supplementary Fig. 1). Additional sequences acquired from GenBank were incorporated into a dataset, which included all syngens also from references known for *P. bursaria*. The phylogenetic analyses revealed five highly supported lineages among the *P. bursaria* strains, which corresponded to their syngen assignment. As demonstrated in Fig. 2, all investigated strains belonging to the syngens R1, R2 and R5 originated from Europe, whereas the others of the syngens R3-R4 showed a worldwide distribution. The three known green algal endosymbionts, i.e., *Chlorella variabilis* (Cvar), *Chlorella vulgaris* (Cvul) and *Micractinium conductrix* (Mcon) showed no or only little affiliation to specific syngens.

### Synapomorphies of the *Paramecium bursaria* Syngens

**Figure 3:**
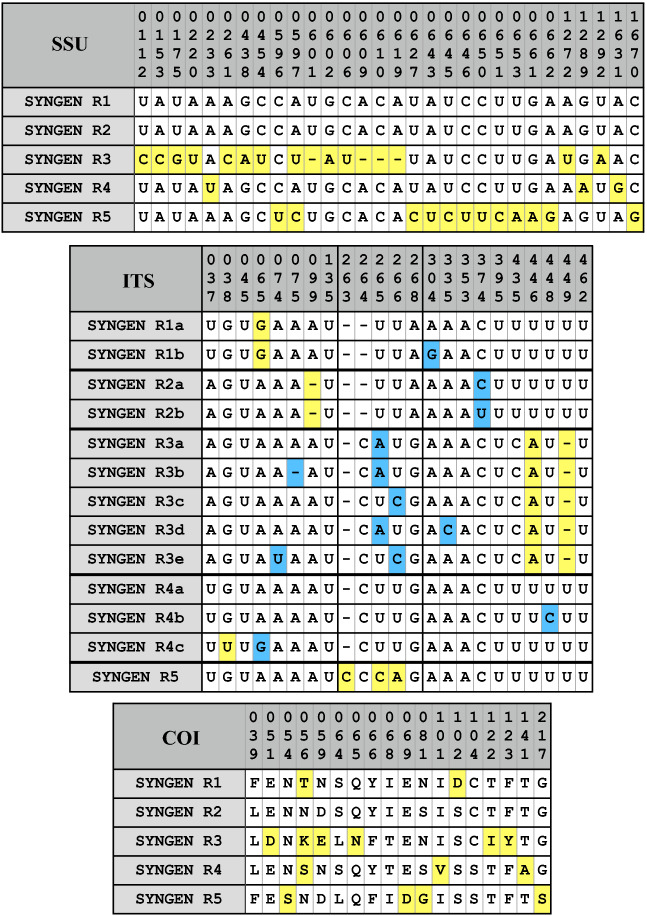
Variable base positions among the SSU, ITS rRNA, and COI sequences of the five syngens among the *Paramecium bursaria* species complex. The unique synapomorphies are highlighted in yellow, variable positions marked in blue.

As demonstrated in Fig. 2, the subdivision of the *P. bursaria* strains into syngens is supported by the phylogenetic analyses of the SSU and ITS rDNA sequences. To figure out if these splits were also supported by characteristic molecular signatures, we studied the secondary structures of both SSU and ITS of all available sequences. We discovered 30, respectively 23 variable positions among the SSU and ITS sequences (numbers of these positions in the respective alignments are given in Fig. 3). All syngens showed characteristic patterns among the SSU and ITS. Only the syngens R1 and R2 could not be distinguished using the SSU only, however, in combination with the ITS, each syngen is characterized by unique synapomorphies as highlighted in yellow (Fig. 3). In addition, few variable base positions within syngens (marked in blue in Fig. 3) have been recognized in the ITS regions. For comparison with literature data, we also analyzed all available sequences of the mitochondrial COI gene to find synapomorphies for the five syngens. Within this gene, only 18 variable positions at the amino acid level could be discovered of which 13 are diagnostic for the five syngens (Fig. 3).

**Figure 4:**
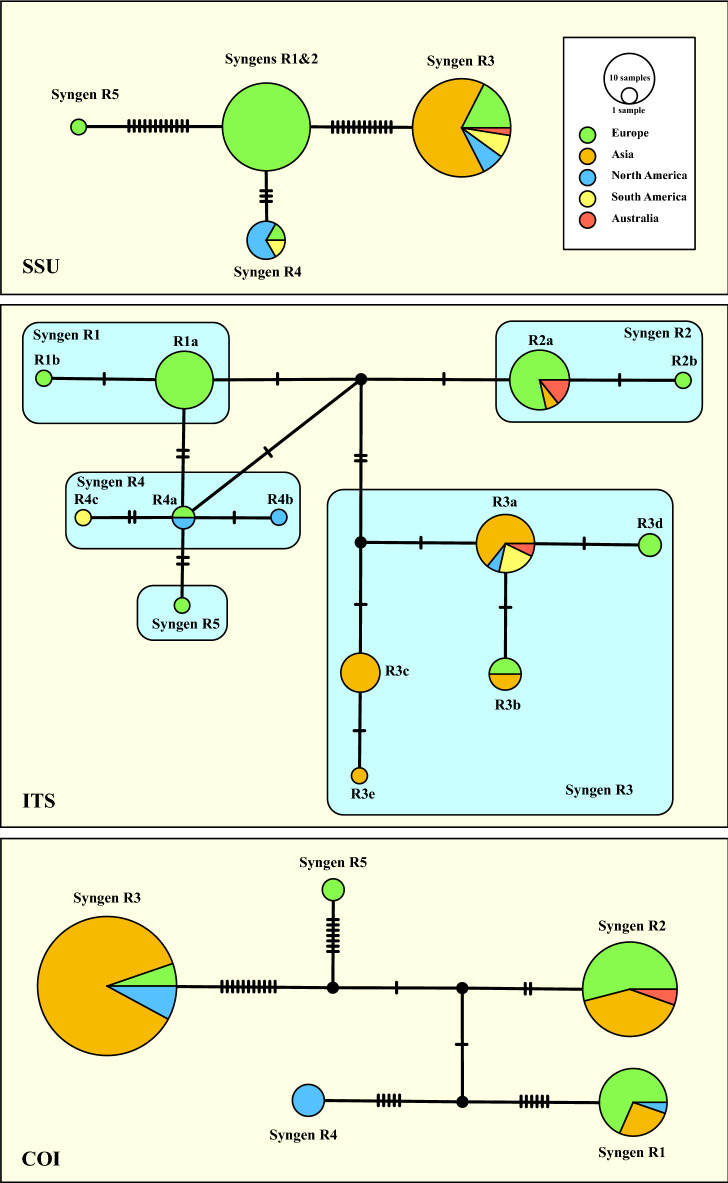
TCS haplotype networks of the five syngens inferred from SSU, ITS rRNA, and COI sequences of the *Paramecium bursaria* species complex. This network was inferred using the algorithm described by Clement et al.^40,41^. Sequence nodes corresponding to samples collected from different geographical regions.

The synapomorphies discovered above were used to get insights into the geographical distribution of each *P. bursaria* syngen. Despite the complete SSU and ITS rDNA sequences included in the phylogeny presented in Fig. 2, records of the partial SSU or ITS rDNA sequences are available in GenBank (BLASTn search; 100% identity;^13^). Considering the metadata of our investigated strains and of the entries in GenBank (Supplementary Table 1), we constructed three haplotype networks using the Templeton-Crandall-Sing (TCS) approach. The SSU haplotype network (Fig. 4) containing 84 records showed that the syngens R1, R2 and R5 were only found in Europe, whereas the other three syngens have been discovered around the world. A similar distribution pattern occurred when using the ITS (101 entries in GenBank). Records of syngens R1 and R5 have only been found in Europe, whereas all other syngens were distributed around the world. The 132 COI records found in GenBank by the BLASTn search were used for the haplotype network, which also showed the similar pattern (Fig. 4).

### Ciliate Taxonomy

**Figure 5:**
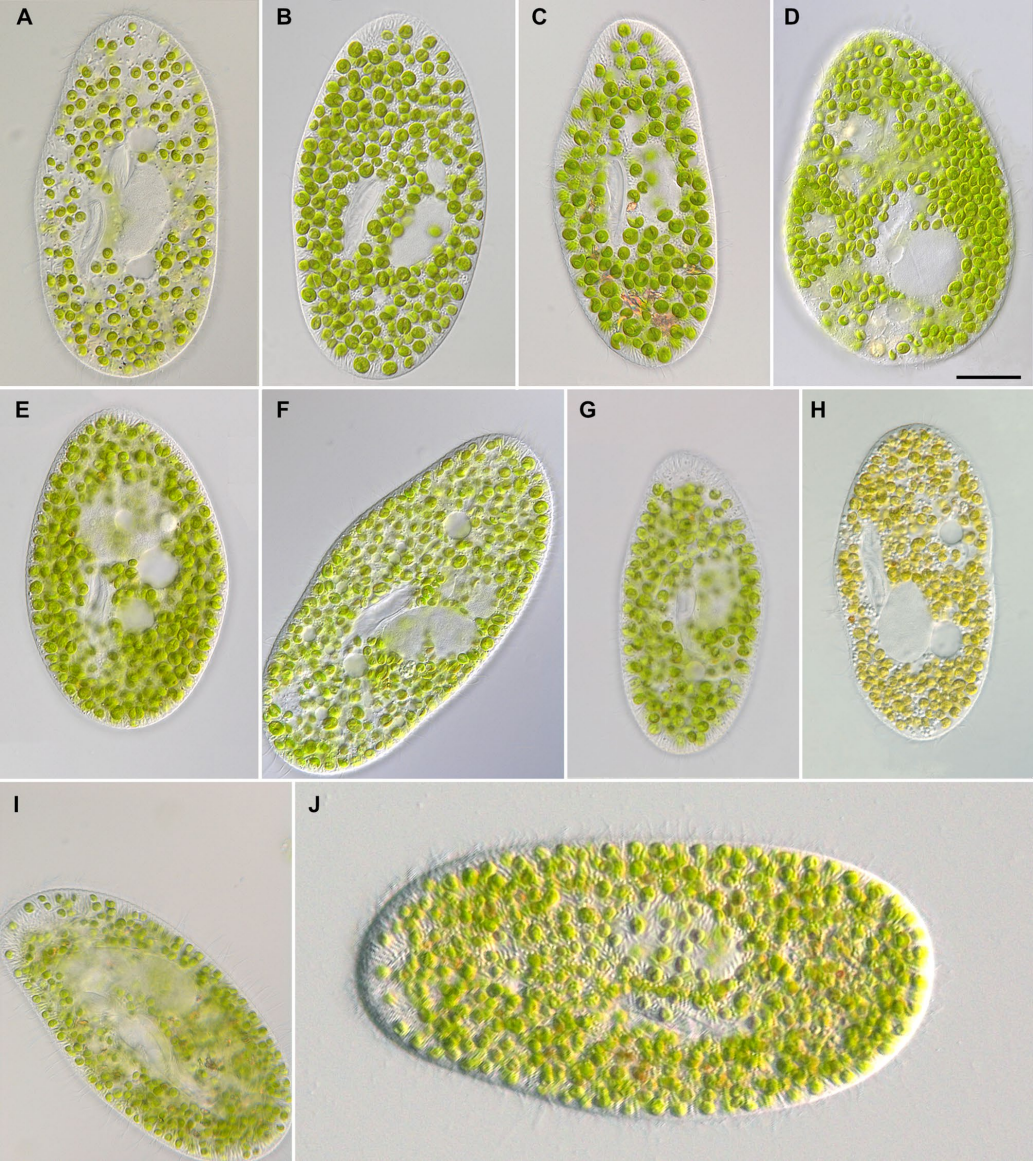
Ventral views of *Paramecium bursaria* morphotypes in vivo: *P. protobursaria* (syngen R1), i.e., strains SAG 2645 (**A**) and PB-25 (**B**); *P. deuterobursaria* (syngen R2), i.e., strains CCAP 1660/36 (**C**) and CCAP 1660/34 (**D**); *P. tritobursaria* (syngen R3), i.e., strains CCAP 1660/28 (**E**), CCAP 1660/26 (**F**) and CCAP 1660/31 (**G**); *P. tetratobursaria* (syngen R4), i.e., strains CCAP 1660/25 (**H**) and CCAP 1660/33 (**I**); *P. pentobursaria* (syngen R5), i.e., strain CCAP 1660/30 (**J**). Scale bar 20 µm.

**Figure 6:**
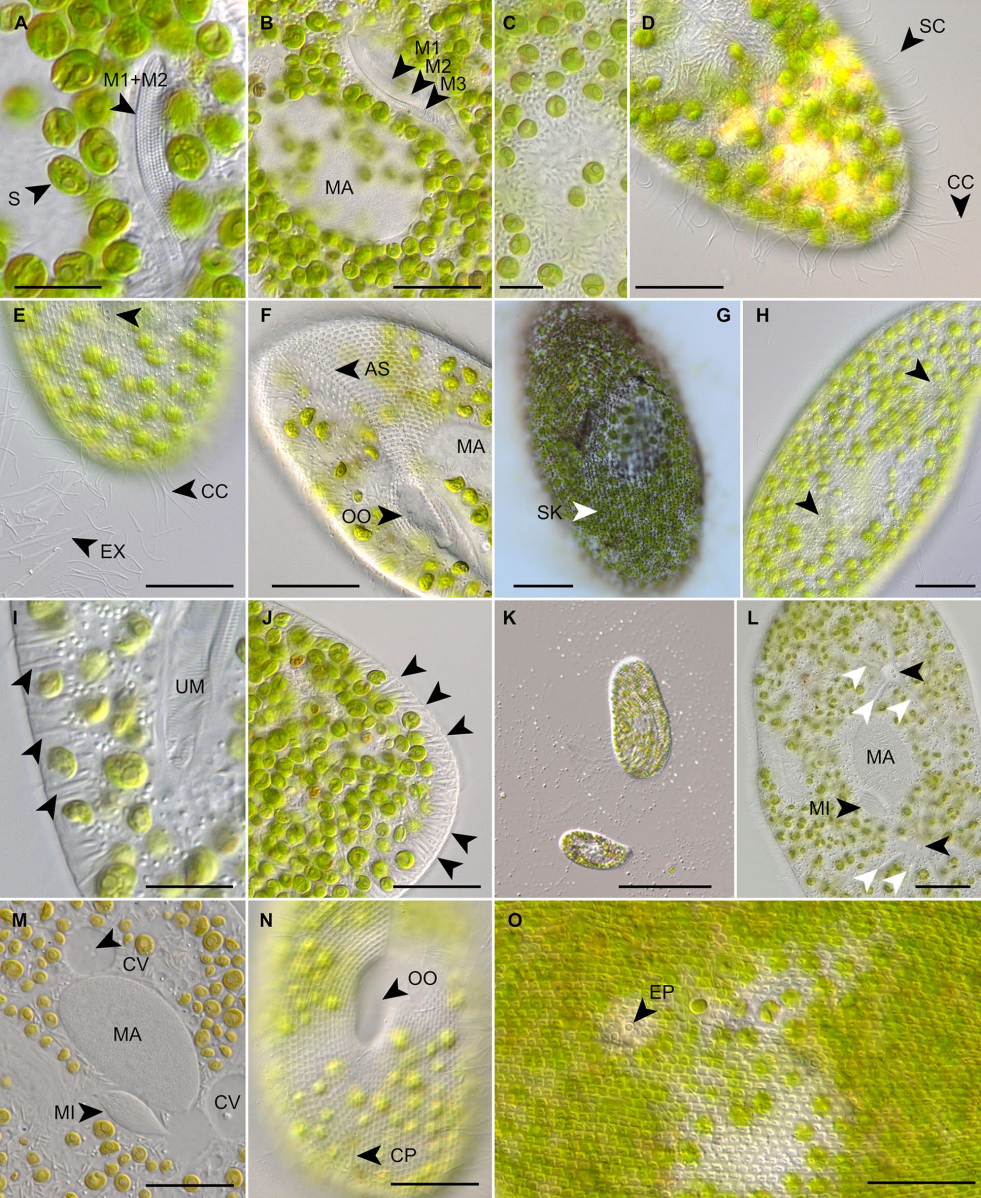
Morphological details of the *Paramecium bursaria* species complex from specimens of strains PB-25 (**A**), CCAP 1660/30 (**B**), SAG 2645 (**C**, **F**, **G**, **I**, **L**–**N**), CCAP 1660/36 (**D**), CCAP 1660/26 (**E**, **H**), CCAP 1660/30 (**J**, **O**), CCAP 1660/16 (**K**) in vivo (**A**–**F**, **H**–**O**) and after silver nitrate staining (**G**). Adoral membranelles (**A**, **B**), endosymbiotic algae *Micractinium conductrix* (**C**), caudal and somatic cilia (**D**), arrows denote excretory pores of the contractile vacuoles: extruded extrusomes are shown and caudal cilia (**E**), ventral views showing the preoral suture and the oral opening (**F**), the ciliary pattern (**G**), arrows denote excretory pores of the contractile vacuoles (**H**), trichocysts and symbiotic algae underneath the pellicula (**I**, **J**), cell size variations (**K**), radial collecting channels (white arrows) and excretory pores (black arrows) of contractile vacuoles (**L**), macro- and micronucleus (**M**), cytopyge and characteristic rectangular pellicular pattern (**N**), pattern of the pellicula (**O**). *AS* anterior suture, *CC* caudal cilia, *CP* cytopyge (cell after), *CV* contractile vacuole, *EP* excretory pore of a contractile vacuole, *EX* extrusomes, *M1–M3* membranelles 1–3, *MA* macronucleus, *MI* micronucleus, *OO* oral opening, *S* symbiotic algae, *SC* somatic cilia, *SK* somatic kineties, *UM* undulating membrane. Scale bars 10 µm (**A**, **I**), 20 µm (**B**, **D**–**H**, **J**, **L**–**O**), 50 µm (**K**).

Considering all our findings, *P. bursaria* is morphologically highly variable, and obviously represents a cryptic species complex (Figs. 5, 6; Supplementary Table 2). The known five syngens most likely represent biological species according to Mayr^14^ and can be attributed to the cryptic species described by Greczek-Stachura et al.^11^. As mentioned above, the assignments of these cryptic species by Greczek-Stachura et al.^11^ have not been validly described according to the ICZN. In addition, the naming using a mixture of Latin prefix and Greek suffix is also not appropriate (the epithet *bursa* derived from the Greek word *byrsa*). Therefore, we describe the five syngens as new species as follows. The general morphological features of these species are summarized in Table 1.

**Table 1:** Main morphometric and morphological characteristics of the *Paramecium bursaria* syngens (min and max values).

	*P. protobursaria*	*P. deuterobursaria*	*P. tritobursaria*	*P. tetratobursaria*	*P. pentobursaria*
Cell shape	Ellipsoidal to broadly ellipsoidal and dorso-ventrally flattened	Ellipsoidal to broadly ellipsoidal and dorso-ventrally flattened	Ellipsoidal to broadly ellipsoidal and dorso-ventrally flattened	Ellipsoidal to broadly ellipsoidal and dorso-ventrally flattened	Ellipsoidal to broadly ellipsoidal and dorso-ventrally flattened
Cell size (µm)	70–164 × 44–65	81–167 × 35–83	80–153 × 49–73	65–179 × 37–79	161–194 × 76–99
MA size (µm)	25–38 × 11–22	24–46 × 10–32	21–53 × 12–31	18–53 × 10–29	24–47 × 19–31
MI type	Compact	Compact	Compact	Compact	Compact
MI size (µm) and *without MI	11–20 × 5–8	10–18 × 5–9*	9–17 × 3–6*	8–18 × 4–10	13–20 × 4–9
# Ciliary rows/20 µm	14–22	13–22	12–20	14–19	13–19
Caudal cilia length (µm)	9–19	11–20	8–19	12–20	14–25
Extrusomes length (µm)	4–6	4–6	4–6	4–7	5–7
# Contractile vacuoles (CV)	2 (rarely 1)	2 (rarely 1 or 3)	2 (rarely 1 or 3)	2 (rarely 1 or 3)	2 (rarely 1 or 3)
Type of contractile vacuoles	"Canal-fed" type with radial canals	"Canal-fed" type with radial canals	"Canal-fed" type with radial canals	"Canal-fed" type with radial canals	"Canal-fed" type with radial canals
# Excretory pores/CV	1–3	1–3	1–3	1–3	1–4
Green algal endosymbiont	*Micractinium conductrix*	*Micractinium conductrix*	*Chlorella variabilis*	*Micractinium conductrix* or *Chlorella variabilis* or *Chlorella vulgaris*	*Chlorella variabilis*

The asterisk indicates that a micronucleus was not seen in live specimens in one of the investigated strains (see Table S2 for details).

### *Paramecium protobursaria* sp. nov.

Synonym: *Paramecium primabursaria* nom. inval.

**Description**: The strains SAG 27.96 and PB-25 belong to syngen R1 according to Greczek-Stachura et al.^10,11^ and differ from other syngens by their SSU and ITS rDNA sequences (MT231333). From morphology, the cells are ellipsoidal to broadly ellipsoidal and dorso-ventrally flattened in vivo. The cells measure 70–164 × 44–65 µm; the single macronucleus is located around mid-cell and measures 25–38 × 11–22 µm; the adjacent single compact micronucleus measures 11–20 × 5–8 µm; the usually two (rarely one) contractile vacuoles, one in the anterior and one in the posterior cell portion have radial collecting channels and 1–3 excretory pores each; the number of ciliary rows/20 µm is 14–22; the length of the caudal cilia is 9–19 µm; the numerous trichocysts located in the cell cortex are 4–6 µm in length. The symbiotic algae belong to *M. conductrix*; the larger algae measure 4–7 × 4–7 µm; the smaller algal cells measure 2–5 × 2–5 µm.

**Geographic distribution**: The investigated strains of syngen R1 were found in Europe: Göttingen, Germany; Lake Mondsee, Austria. In addition, this species has been reported from different places in Europe, Asia and North America (see details in Supplementary Table 1).

**Reference material**: Strain SAG 27.96 and the clonal strain SAG 2645 derived from SAG 27.96 are available at the Culture Collection of Algae (SAG), University of Göttingen, Germany.

**Holotype**: Two slides (one holotype, one paratype) with protargol-impregnated specimens from the clonal culture SAG 2645, which derived from the reference material SAG 27.96, isolated from the pond of the Old Botanical Garden of the University of Göttingen (Germany), have been deposited in the Oberösterreichisches Landesmuseum at Linz (LI, Austria).

**Zoobank Registration LSID**: AFD967ED-BC2A-43FD-847E-5DF588BB025C.

### *Paramecium deuterobursaria* sp. nov.

Synonym: *Paramecium bibursaria* nom. inval.

**Description**: The strains CCAP 1660/34 and CCAP 1660/36 belong to syngen R2 according to Greczek-Stachura et al.^10,11^ and differ from other syngens by their SSU and ITS rDNA sequences (OK318487). From morphology, the cells are ellipsoidal to broadly ellipsoidal and dorso-ventrally flattened in vivo. The cells measure 81–167 × 35–83 µm; the single macronucleus is located around mid-cell and measures 24–46 × 10–32 µm; the adjacent single compact micronucleus measures 10–18 × 5–9 µm, no micronucleus seen in live cells of strain CCAP 1660/34; the usually two (rarely one or three) contractile vacuoles, one in the anterior and one in the posterior cell portion have radial collecting channels and 1–3 excretory pores each; the number of ciliary rows/20 µm is 13–22; the length of the caudal cilia is 11–20 µm; the numerous trichocysts located in the cell cortex are 4–6 µm in length. The symbiotic algae belong to *M. conductrix*; the larger algae measure 5–7 × 4–7 µm; the smaller algal cells measure 3–5 × 2–5 µm.

**Geographic distribution**: The investigated strains of syngen R2 were found in Europe: Zurich, Switzerland; Lake Piburg, Austria. In addition, this species has been reported from different places in Europe, Asia and Australia (see details in Supplementary Table 1).

**Reference material**: Strain CCAP 1660/36 is available at the Culture Collection of Algae and Protozoa (CCAP) at the Scottish Association for Marine Science, Oban, Scotland.

**Holotype**: Two slides (one holotype, one paratype) with protargol-impregnated specimens from the reference material CCAP 1660/36, isolated from Lake Piburg (Tyrol, Austria), have been deposited in the Oberösterreichisches Landesmuseum at Linz (LI, Austria).

**Zoobank Registration LSID**: D1C20BE6-9A15-4A3D-A7E5-DFC31FF04679.

### ***Paramecium tritobursaria*** sp. nov.

Synonym: *Paramecium tribursaria* nom. inval.

**Description**: The strains CCAP 1660/26, CCAP 1660/28 and CCAP 1660/31 belong to syngen R3 according to Greczek-Stachura et al.^10,11^ and differ from other syngens by their SSU and ITS rDNA sequences (MT231339). From morphology, the cells are ellipsoidal to broadly ellipsoidal and dorso-ventrally flattened in vivo. The cells measure 80–153 × 49–73 µm; the single macronucleus is located around mid-cell and measures 21–53 × 12–31 µm; the adjacent single compact micronucleus measures 9–17 × 3–6 µm; no micronucleus seen in live cells of strain CCAP 1660/28; the usually two (rarely one or three) contractile vacuoles, one in the anterior and one in the posterior cell portion have radial collecting channels and 1–3 excretory pores each; the number of ciliary rows/20 µm is 12–20; the length of the caudal cilia is 8–19 µm; the numerous trichocysts located in the cell cortex are 4–6 µm in length. The symbiotic algae belong to *C. variabilis*; the larger algae measure 4–7 × 3–6 µm; the smaller algal cells measure 3–5 × 2–4 µm.

**Geographic distribution**: The investigated strains of syngen R3 were found in Europe and Asia: Lake Piburg, Austria; Tokyo, Japan; Khabarovsk region, Amur River, Russia. In addition, this species has been reported from different places in Europe, Asia, North and South America as well as in Australia (see details in Supplementary Table 1).

**Reference material**: Strain CCAP 1660/26 is available at the Culture Collection of Algae and Protozoa (CCAP) at the Scottish Association for Marine Science, Oban, Scotland.

**Holotype**: Two slides (one holotype, one paratype) with protargol-impregnated specimens from the reference material CCAP 1660/26, isolated from Japan, have been deposited in the Oberösterreichisches Landesmuseum at Linz (LI, Austria).

**Zoobank Registration LSID**: CC0FBA7E-9E3A-4C37-B424-C9BFF2018EC0.

### ***Paramecium tetratobursaria*** sp. nov.

Synonym: *Paramecium tetrabursaria* nom. inval.

**Description**: The strains CCAP 1660/25 and CCAP 1660/33 belong to syngen R4 according to Greczek-Stachura et al.^10,11^ and differ from other syngens by their SSU and ITS rDNA sequences (MT231347). From morphology, the cells are ellipsoidal to broadly ellipsoidal and dorso-ventrally flattened in vivo. The cells measure 65–179 × 37–79 µm; the single macronucleus is located around mid-cell and measures 18–53 × 10–29 µm; the adjacent single compact micronucleus measures 8–18 × 4–10 µm; the usually two (rarely one or three) contractile vacuoles, one in the anterior and one in the posterior cell portion have radial collecting channels and 1–3 excretory pores each; the number of ciliary rows/20 µm is 14–19; the length of the caudal cilia is 12–20 µm; the numerous trichocysts located in the cell cortex are 4–7 µm in length. The symbiotic algae belong to *C. variabilis* (CCAP 1660/25) and *M. conductrix* (CCAP 1660/33); the larger algae measure 3–6 × 3–6 µm; the smaller algal cells measure 2–5 × 1–4 µm.

**Geographic distribution**: The investigated strains of syngen R4 are found in North- and South America: Burlington, North Carolina, USA; San Pedro de la Paz, Laguna Grande, Chile. In addition, this species has been reported from Europe (see details in Supplementary Table 1).

**Reference material**: Strain CCAP 1660/25 is available at the Culture Collection of Algae and Protozoa (CCAP) at the Scottish Association for Marine Science, Oban, Scotland.

**Holotype**: Two slides (one holotype, one paratype) with protargol-impregnated specimens from the reference material CCAP 1660/25, isolated from a pond in Burlington (North Carolina, USA), have been deposited in the Oberösterreichisches Landesmuseum at Linz (LI, Austria).

**Zoobank Registration LSID**: 78BA9923-07A9-4918-AD7C-9E5E15CC9CDB.

### ***Paramecium pentobursaria*** sp. nov.

Synonym: *Paramecium pentabursaria* nom. inval.

**Description**: The strain CCAP 1660/30 belongs to syngen R5 according to Greczek-Stachura et al.^10,11^ and differs from other syngens by their SSU and ITS rDNA sequences (MT231348). From morphology, the cells are ellipsoidal to broadly ellipsoidal and dorso-ventrally flattened in vivo. The cells measure 161–194 × 76–99 µm; the single macronucleus is located around mid-cell and measures 24–47 × 19–31 µm; the adjacent single compact micronucleus measures 13–20 × 4–9 µm; the usually two (rarely one or three) contractile vacuoles, one in the anterior and one in the posterior cell portion have radial collecting channels and 1–4 excretory pores each; the number of ciliary rows/20 µm is 13–19; the length of the caudal cilia is 14–25 µm; the numerous trichocysts located in the cell cortex are 5–7 µm in length. The symbiotic algae belong to *C. variabilis*; the larger algae measure 5–6 × 5–6 µm; the smaller algal cells measure 4–5 × 3–4 µm.

**Geographic distribution**: The investigated strain of Syngen R5 was found in Europe: Astrakhan Nature Reserve, Russia.

**Reference material**: Strain CCAP 1660/30 is available at the Culture Collection of Algae and Protozoa (CCAP) at the Scottish Association for Marine Science, Oban, Scotland.

**Holotype**: Two slides (one holotype, one paratype) with protargol-impregnated specimens from the reference material CCAP 1660/30, isolated from Astrakhan Nature Reserve (Russia), have been deposited in the Oberösterreichisches Landesmuseum at Linz (LI, Austria).

**Zoobank Registration LSID**: 6629FA71-E00F-48C6-83AB-61C0CA4823B6.

### Syngen Affiliation related to Ciliate Morphology, Endosymbionts and Geographic Distribution

**Figure 7:**
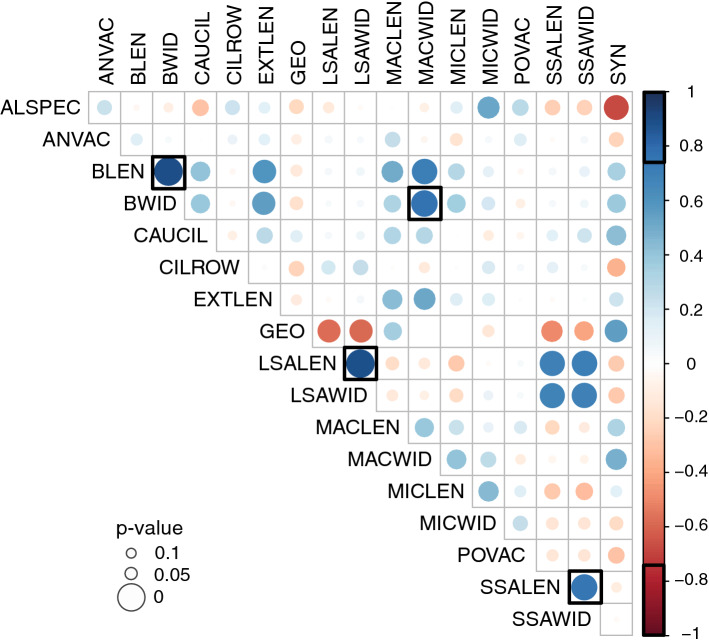
Pearson-correlations of morphometric, symbiont and syngen data of *Paramecium* strains under study. Colored dots indicate the strength of correlation, and the size of dots represent p-values. Bold squares highlight significant correlations, with − 0.75 > r > 0.75 and p < 0.05. Abbreviations: *ANVAC* number of excretory pores in anterior contractile vacuole, *ALSPEC* algal species, *BLEN* body/cell length, *BWID* body/cell width, *CAUCIL* caudal cilia length, *CILROW* number of ciliary rows, *EXTLEN* extrusome/trichocyst length, *GEO* geographical region, *LSALEN* large symbiotic algae length, *LSAWID* large symbiotic algae width, *MACLEN* macronucleus length, *MACWID* macronucleus width, *MICLEN* micronucleus length, *MICWID* micronucleus width, *POVAC* number of excretory pores in posterior contractile vacuole, *SSALEN* small symbiotic algae length, *SSAWID* small symbiotic algae width, *SYN* syngen affiliation.

Pearson-correlations of morphometric, syngen-specific and endosymbiont datasets of the *P. bursaria* strains revealed four significant positive correlations (p < 0.05 and − 0.75 > r > 0.75) between ciliate cell length (BLEN) and width (BWID), BWID and macronucleus width (MACWID), as well as length and width of large symbiotic algae (LSALEN and LSAWID; Fig. 7).

**Figure 8:**
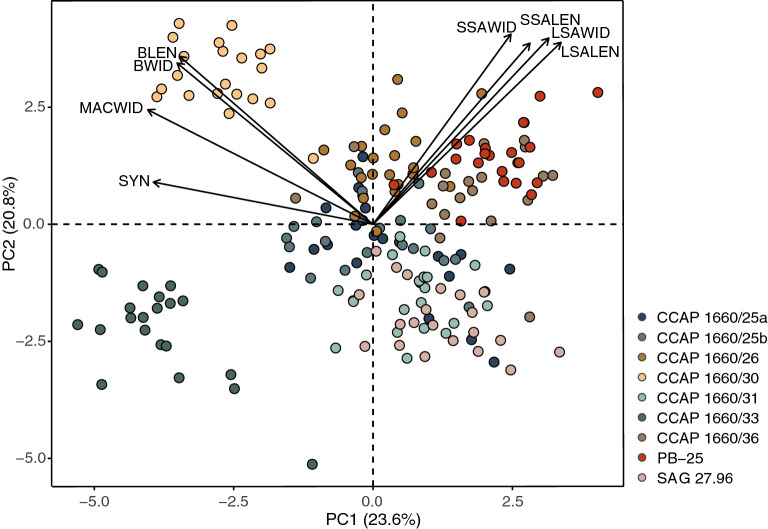
PCA of morphometric data of *Paramecium bursaria* strains. Only the top eight contributing variables are shown.

The results of the principal component analysis (PCA) are summarized in the ordination diagram in Fig. 8. The first two axes explain 44.4% of the total variation in the investigated features. Only the first five components (out of 18) had eigenvalues > 1, accounting for 73.1% variation in total (Supplementary Table 3). Principal component axis 1 (PC1) appears to be most negatively weighted by syngen (SYN) and width of the macronucleus (MACWID), separating CCAP 1660/30 and CCAP 1660/33 from the other strains. Principal component axis 2 (PC2) is primarily positively influenced by symbiotic algae characteristics (LSALEN, LSAWID, small symbiotic algal length (SSALEN) and width (SSAWID)) and, ciliate cell length (BLEN) and width (BWID; Supplementary Table 4), partitioning strain PB-25, CCAP 1660/26 and CCAP 1660/36 from CCAP 1660/31 and SAG 27.96 (Fig. 8).

**Figure 9:**
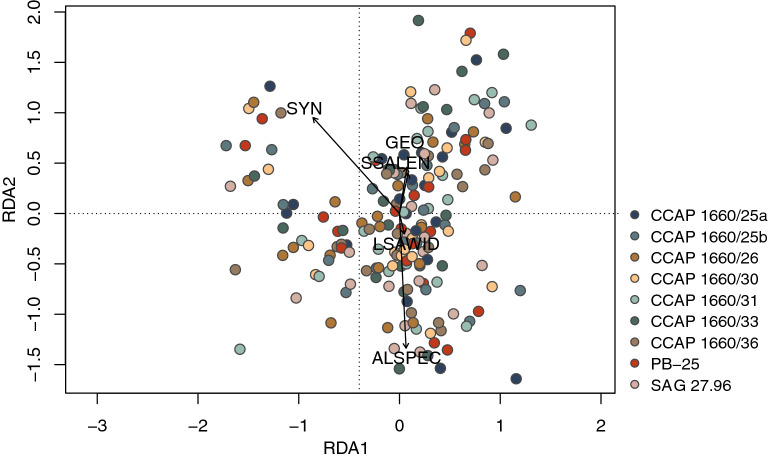
Ordination diagram for redundancy analysis (RDA) of morphometric data and shown syngen (SYN), geographic region (GEO), and algal features (ALSPEC, LSAWID and SSALEN) as explanatory features.

The redundancy analysis (RDA; Fig. 9) revealed a large difference between morphometric features and the tested set of explanatory variables (i.e., algal species (ALSPEC), LSAWID, SSALEN, SYN and GEO) as only 26.9% of the total variation could be explained.

## Discussion

Among strains of *P. bursaria*, six syngens have been discovered so far by mating experiments^5,9,10^. Our phylogenetic analyses using a concatenated dataset of SSU and ITS sequences revealed five highly supported lineages among the investigated *P. bursaria* strains, which clearly corresponded to the cryptic species assigned to syngens R1-R5 according to Greczek-Stachura et al.^10,11^. All syngens could be individually distinguished by their molecular signatures (Fig. 3), however, isolates belonging to syngens R1 and R2 could not be recognized by sequencing their SSU rDNA only.

*Paramecium bursaria* are distributed worldwide (Fig. 4). Only the syngens R1 and R5 have been found in Europe, whereas the other syngens have been recorded from Europe, Asia, North and South America and Australia. However, very little is known from other regions of the world such as South America, Australia or Africa.

The available strains of *P. bursaria* were mostly isolated for studying their green algal endosymbionts. Originally, these endosymbionts were differentiated into two groups: an American (or Southern) and a European (or Northern) type^15–20^. Pröschold et al.^1^ taxonomically revised both groups and emended the description of the two species *C. variabilis* and *M. conductrix* based on the authentic strains (SAG 211–6 and SAG 241.80). Considering both of these strains, Spanner et al.^21^ developed an easy diagnostic PCR approach for the isolation and identification of the *zoochlorellae* living in *P. bursaria* revealing that both endosymbiotic species were found in almost all syngens (Fig. 2). In syngen R3, only *C. variabilis* was detected. Interestingly, in the strain CCAP 1660/10, belonging to syngen R4, *C. vulgaris* has been reported^19^. The assignment to syngen R4 of this strain is surprising because this is the only record from Europe. This has to be taken with caution because the assignment of another strain CCAP 1660/11 to syngen R5 by Hoshina & Imamura^19^ is incorrect as demonstrated in our study. This strain belongs to syngen R1 (Fig. 2; Table S1). Unfortunately, this strain is lost and the syngen assignment cannot be proven. *Chlorella vulgaris* occurred either free-living or as endosymbiont of ciliates such as *Euplotes daidaleos*, *Coleps hirtus*, *Climacostomum virens* and *P. bursaria*^1^. Unfortunately, those ciliates are neither available in public culture collections nor their molecular datasets in public databases. Consequently, the ciliate host/syngen from which *C. vulgaris* had originally been isolated remains unknown. Recently, Greczek-Stachura et al.^22^ reported that *Chlorella sorokiniana*, a free-living species from warm-temperate habitats, also occurred in three isolates of *P. bursaria* collected in Lake Baikal and the Kamchatka region (Asian part of Russia). The investigations were based on the partial nuclear large subunit (LSU) rDNA and chloroplast genes encoding the ribosomal protein L36 (*rpl*36) and translation initiation factor IF-1 (*inf*A). Unfortunately, no ITS of these isolates has been sequenced and, accordingly, the assignment to *C. sorokiniana* is questionable. For example, the Chinese *P. bursaria* strain Cs2 (R3) bears the “American” type of endosymbiont as demonstrated by Hoshina et al.^16^, which is *C. variabilis* and not *C. sorokiniana*^1^. Moreover, two other reports of Greczek-Stachura et al.^22^ were probably incorrect: the strains AZ20-1 (according to CCAP 1660/30 in our study; R5) and Yad1-g (R3), both have *C. variabilis*, and not *C. vulgaris* as endosymbiont^21,23^ (this study; see also Supplementary Table 1). Both molecular markers (partial LSU, *rpl*36-*inf*A) do not have the diagnostic power for a discrimination of green algal endosymbionts at the species level.

Ciliate descriptions and taxonomic assignments basically require the detailed study of species-specific diagnostic features, relevant literature, and biogeographical aspects^24^. It is consequently necessary that molecular and microscopic approaches are closely linked for a certain population or strain, especially when the ciliate’s ecology is in the focus of a study^25,26^. Nevertheless, as molecular approaches are becoming major tools in ciliate ecology, the morphological identification of a ciliate still remains hidden in many cases^27^. Since the first description by Ehrenberg^28^, *P. bursaria* was often identified only by the presence of green algal endosymbionts despite reported findings of free-living and naturally algal-free individuals^29^. Moreover, the symbiotic algae can be artificially ‘removed’ from *P. bursaria* for experimental approaches^30,31^. Detailed morphological investigations of this species were lacking for a long time under the assumption that all ‘green’ paramecia were assignable to *P. bursaria*. Kreutz et al.^12^ provided a detailed description on a population of *P. bursaria* and another green congener, *P. chlorelligerum*, a rare species that was originally established by Kahl^32^. Despite Kalmus^33^ already mentioned a high variability of especially the cell shape among *Paramecium* species, very little is known about their phenotypic plasticity. However, from our detailed morphometric analyses of the studied strains, we can confirm that the morphological features unequivocally revealed *P. bursaria* and showed that the characteristics tended to be highly variable (Supplementary Table 2) as already reported by Foissner et al.^34^ in their identification key.

## Conclusions

The *P. bursaria* species complex is widely distributed around the world. As shown, sequencing and analyzing of the SSU and ITS rDNA of isolated samples and strains can clearly assign them to the syngen level. The five lineages revealed by our phylogenetic analyses clearly corresponded to the syngen affiliations. Unfortunately, the syngens could not be identified by morphology only. Further studies are needed to get more insights about the geographical distribution of the *P. bursaria* species complex and its endosymbionts, which both can be clearly determined using our molecular tools presented here. The usage of diagnostic PCR approach provided an easy method for identification of the green algal endosymbionts.

## Methods

### Origin of the investigated strains and cultivation of ciliates and their endosymbionts

The origin of the investigated *P. bursaria* strains is summarized in Table 2. As the respective strains preferred different media, we used modified Bold Basal Medium (3N-BBM + V; medium 26a in Schlösser^35^) with the addition of 30 ml of soil extract per liter (S/BBM; see Spanner et al.^21^), modified Woods Hole MBL (WC) medium^36^ mixed with Volvic® (V) mineral water, in various concentrations, V/WC 1:1, and V/WC 5:1 v/v. All cultures were maintained at 15–21 °C under a light:dark cycle of 12:12 h (photon flux rate up to 50 μmol m^−2^ s^−1^). The isolated green algal endosymbionts were cultivated under the same culture conditions in Basal Medium with beef extract (ESFl; medium 1a in Schlösser^37^).

**Table 2:** List of the investigated *Paramecium bursaria* strains including information on the respective syngen, origin, the green algal endosymbiont and accession number (new accessions highlighted in bold).

Strain #	Syngen	Origin	Accession #	Endosymbiont	Identification method
PB-19	R1 (B6)	Poland: Biebrza National Park	MT231330	Cvar	[1]
CCAP 1660/11	R1 (B6)	England: Cambridge, Cavendish Pond	MT231331	Mcon	[1]
CCAP 1660/12	R1 (B6)	England: Cambridge, Cavendish Pond	MT231332	Mcon	[1], [2]
SAG 27.96	R1 (B6)	Germany: Göttingen, pond in Old Botanical Garden	MT231333	Mcon	[1], [2], [3]
CCAP 1660/46	R1 (B6)	Germany: Göttingen, pond in Old Botanical Garden	**OK318475**	Mcon	[1]
CCAP 1660/47	R1 (B6)	Germany: Göttingen, pond in Old Botanical Garden	**OK318476**	Mcon	[1]
CCAP 1660/37	R1 (B6)	Germany: Pond near Minister	**OK318477**	Mcon	[1]
CCAP 1660/38	R1 (B6)	Germany: Pond near Minster	**OK318478**	Mcon	[1]
PB-25	R1 (B6)	Austria: Lake Mondsee	**OK318479**	Mcon	[1]
CCAP 1660/1B	R1 (B6)	England: Cambridgeshire, coprolite pits Haslingfield	**OK318480**	Mcon	[1]
CCAP 1660/13	R1 (B6)	England: Cambridge, Cavendish Pond	**OK318475**	Mcon	[1]
CCAP 1660/39	R2 (B4)	Germany: Lake Seeburg near Gottingen	MT231334	Cvar	[1]
CCAP 1660/16	R2 (B4)	Scotland: Loch Inverawe, Inverawe	MT231335	Mcon	[1]
CCAP 1660/18	R2 (B4)	Scotland: Loch Lily, Inverawe	MT231336	Mcon	[1]
CCAP 1660/20	R2 (B4)	Scotland: Loch Lily, Inverawe	MT231337	Mcon	[1]
PB-27	R2 (B4)	Austria: Lake Neusiedl	**OK318482**	unknown	–
PB-28	R2 (B4)	Austria: Lake Neusiedl	**OK318483**	unknown	–
CCAP 1660/17	R2 (B4)	Scotland: Loch Inverawe, Inverawe	**OK318484**	Mcon	[1]
CCAP 1660/19	R2 (B4)	Scotland: Loch Lily, Inverawe	**OK318485**	Mcon	[1]
CCAP 1660/34	R2 (B4)	Switzerland: small pond near Zurich	**OK318486**	Mcon	[1]
CCAP 1660/36	R2 (B4)	Austria: Lake Piburg	**OK318487**	Mcon	[1], [2], [3]
PB-26	R2 (B4)	England: Pond near Manchester	**OK318488**	Mcon	[1]
CCAP 1660/24	R2 (B4)	Scotland: Garden pond Kenmore Cottage, Bonawe	**OK318489**	Mcon	[1]
PB-2	R3 (B1)	USA: Massachusetts, Boston	MT231338	Cvar	[1]
CCAP 1660/28	R3 (B1)	Austria: Lake Piburg	MT231339	Cvar	[1], [2], [3]
CCAP 1660/29	R3 (B1)	Austria: Wildbichl	MT231340	Cvar	[1], [2]
CCAP 1660/21	R3 (B1)	Chile: Concepcion, artificial pond at University campus	MT231341	Cvar	[1]
CCAP 1660/22	R3 (B1)	Chile: Concepcion, artificial pond at University campus	MT231342	Cvar	[1]
CCAP 1660/23	R3 (B1)	Chile: Concepcion, artificial pond at University campus	MT231343	Cvar	[1]
CCAP 1660/31	R3 (B1)	Russia: Khabarovsk region, Amur river	MT231344	Cvar	[1]
CCAP 1660/32	R3 (B1)	Russia: Primorie, Kiparisovo	MT231345	Cvar	[1]
PB-17	R3 (B1)	Japan	**OK318490**	Cvar	[1]
CCAP 1660/26	R3 (B1)	Japan	**OK318491**	Cvar	[1]
PB-15	R3 (B1)	Japan	**OK318492**	Cvar	[1]
CCAP 1660/35	R3 (B1)	Japan: Matsue-city Shimane prefecture	**OK318493**	Cvar	[1]
PB-16	R3 (B1)	Japan	**OK318494**	Cvar	[1]
CCAP 1660/27	R3 (B1)	Japan	**OK318495**	Cvar	[1]
PB-11	R3 (B1)	Russia: St. Petersburg	**OK318496**	Cvar	[1]
PB-13	R3 (B1)	Italy: Pisa	**OK318497**	Cvar	[1]
MRBG1	R3 (B1)	Australia: Melbourne	AB219526 + AB252014	Cvar	[2]
So13	R3 (B1)	Japan: Nagano	AB206538 + AB252011	Cvar	[2]
OK1	R3 (B1)	Japan: Aich	AB206537 + AB252010	Cvar	[2]
Cs2	R3 (B1)	China: Shanghai	AB206543 + AB252013	Cvar	[2]
PB-1	R4 (B2)	USA: Massachusetts, Boston	MT231346	Unknown	–
CCAP 1660/25	R4 (B2)	USA: North Carolina, Burlington	MT231347	Cvar	[1], [2], [3]
CCAP 1660/33	R4 (B2)	Chile: Concepcion, Laguna Grande	**OK318498**	Mcon	[1]
CCAP 1660/10	R4 (B2)	England: Cambridge	AB252000 + AB252016	Cvul	[2]
CCAP 1660/30	R5	Russia: Astrakhan Nature Reserve	MT231348	Cvar	[1]

[1] = green algal endosymbiont identified using the diagnostic PCR approach introduced by Spanner et al.^21^.

[2] = identified by direct sequencing.

[3] = identified using the isolation method introduced by Spanner et al.^21^ and sequencing.

Cvar = *Chlorella variabilis*, Cvul = *Chlorella vulgaris*, Mcon = *Micractinium conductrix.*

### DNA extraction, PCR and sequencing

Genomic DNA of the *P. bursaria* strains was extracted using the DNeasy Plant Mini Kit (Qiagen GmbH, Hilden, Germany). The SSU and ITS rDNA were amplified using the Taq PCR Mastermix Kit (Qiagen GmbH, Hilden, Germany) with the primers EAF3 and ITS055R as described in Spanner et al.^21^. The datasets generated and analyzed during the current study are available in GenBank (https://www.ncbi.nlm.nih.gov). The GenBank accession numbers are given in Table 2.

### Identification of the green algal endosymbionts

The green algal endosymbionts were identified using three different approaches: (i) the diagnostic PCR approach^21^, (ii) direct sequencing using the green algal specific primers G500F and G800R as described by Darienko et al.^38^, and (iii) isolation using the method introduced by Spanner et al.^21^ and sequencing of the SSU and ITS rDNA with the green algal specific primers. The respective identification method used is given in Table 2.

### Phylogenetic and network analyses

All sequences were aligned to their secondary structures as demonstrated for strain SAG 27.96 (Fig. 1; Supplementary Fig. 1). The secondary structures were folded using the software *mfold*^39^, which uses the thermodynamic model (minimal energy) for RNA folding. The visualization of the structures was manually done using the program Illustrator CS5.1 (Adobe Inc.). For the phylogenetic analyses, we calculated the log-likelihood values of 56 models using the automated selection tool implemented in PAUP version 4.0b169^40^ to test which evolutionary model fit best for the dataset. The best model according to the Akaike criterion by PAUP was chosen. The settings of the best model were given in the figure legends. The following methods were used for the phylogenetic analyses: distance, maximum parsimony, and maximum likelihood, all included in PAUP version 4.0b169^40^.

The secondary structures of the SSU and ITS rRNA sequences were compared to find genetic synapomorphies, which were used for the construction of haplotype networks. To establish an overview on the distribution of each syngen, the SSU and ITS haplotypes were used for a BLASTn search (100% coverage, > 97% identity; Altschul et al.^13^). To construct the haplotype networks, we used the Templeton-Crandall-Sing (TCS) network tool^41,42^ implemented in PopART^43^. The COI sequences presented in Greczek-Stachura et al.^10,11^ were analyzed to find synapomorphies at the amino acid level.

### Morphological investigations of ciliates and endosymbionts

The morphology of the *P. bursaria* strains and their endosymbionts was studied mainly from living individuals, which were cloned using the isolation method (steps 1 and 2) described in Spanner et al.^21^. After 24 h of starvation, the single ciliate cells were cultivated in 24-well plates (Biomedica) each in the cultivation media mentioned above. To reveal their ciliary pattern, additionally, a dry silver nitrate impregnation was applied^44^. All protists were studied under bright field and differential interference contrast optics with an Olympus BX51 and an Olympus BX60 microscope (Olympus, Vienna, Austria) with 40–1000 × magnifications. For documentation and measurements, two digital image analysis systems were used (ProgRes SpeedXT core 5 2.9.0.1. and ProgRes Capture Pro imaging system version 2.9.0.1, Jenoptik, Jena, Germany). The ciliates were identified by means of the key of Foissner et al.^34^ and Kreutz et al.^12^ and standard morphometric calculations were done. The green algae were identified by comparison with the descriptions presented in Pröschold et al.^1^. Type slides (holotypes, paratypes) were stained with protargol (Skibbe method)^45^.

### Multivariate analyses of morphometric, symbiont and syngen data of *Paramecium* strains

All correlation and multivariate analyses were conducted in R version 4.1.1 using the *stats* and *vegan* packages. Statistical analyses included all morphometric, syngen and geographic origin information, as well as algal symbiont features of the *Paramecium* strains under study (Figs. 7, 8, 9). Strains CCAP 1660/28 and CCAP 1660/34 were excluded from downstream analyses as no micronucleus data (= no micronucleus could be seen in the ciliates) were available.

All data were first checked for normality with a Shapiro–Wilk test and then used to run standard Pearson correlations between each other to rule out any correlations. Correlations were considered significant if p < 0.05 and − 0.75 > r > 0.75. The overall variation in the dataset was summarized with a PCA (unconstrained ordination). The relationship between morphometric features (response variables) and explanatory variables, representing syngen and symbiont features, was summarized using an RDA (constrained ordination) with centered data. Features GEO, LSALEN and SSAWID were removed from analysis due to multicollinearity with SYN, LSAWID and SSALEN, respectively (Supplementary Tables 3–4). The significance of the observed relationship was tested with a Monte Carlo permutation test using 999 permutations.

## Supplementary Information

Supplementary Figures.

Supplementary Table 1.

Supplementary Table 2.

Supplementary Table 3.

Supplementary Table 4.
