# Supplementary figures and images for: Morphological diversity and molecular phylogeny of five Paramecium bursaria (Alveolata, Ciliophora, Oligohymenophorea) syngens and the identification of their green algal endosymbionts
Source: Sci Rep. 2022 Oct 27;12:18089. doi: 10.1038/s41598-022-22284-z (PMC9613978; doi:10.1038/s41598-022-22284-z)

SSU rRNA secondary structure model  
of *Paramecium protobursaria*  
(SAG 27.96)  
Accession number: MT231333

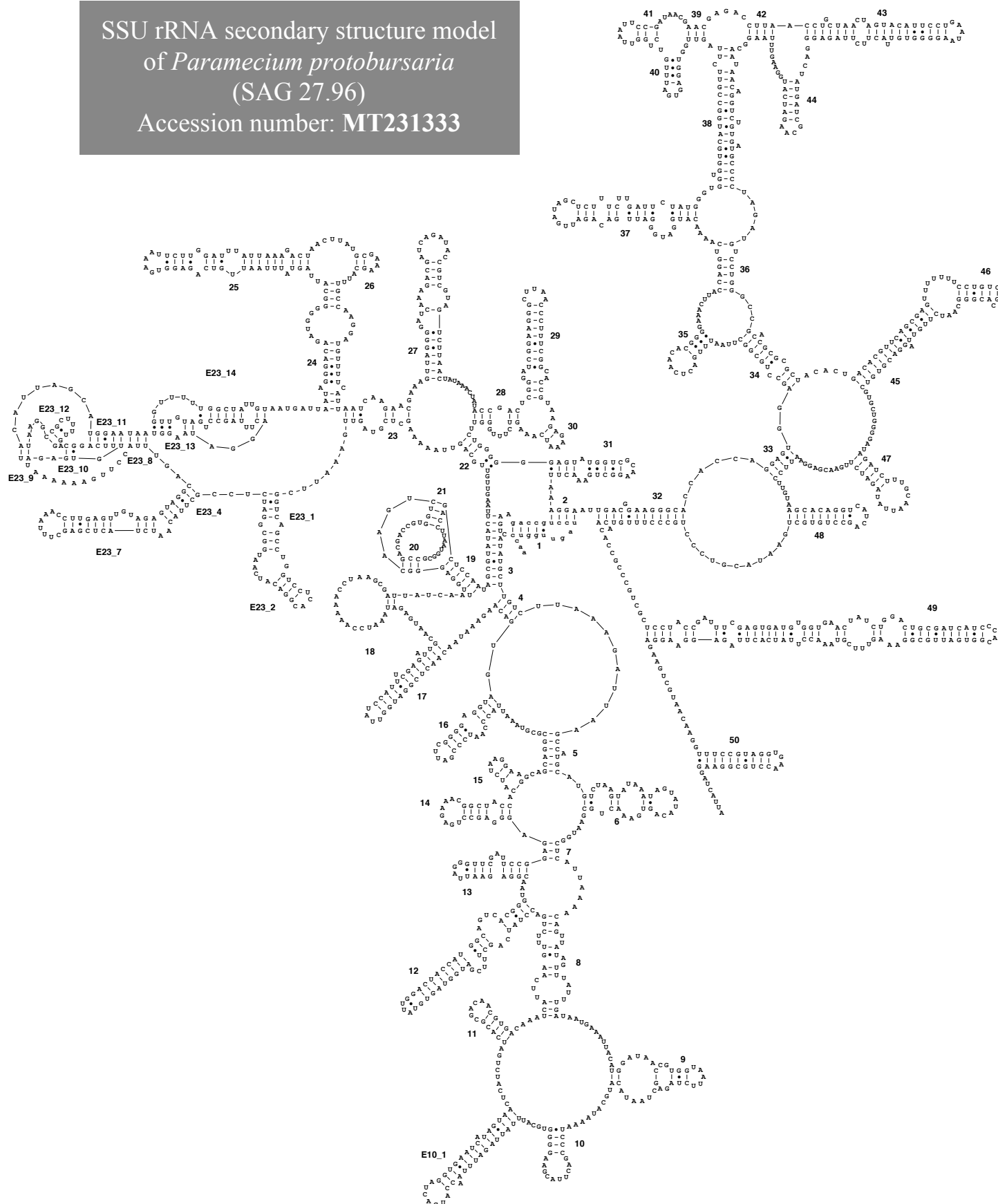

Supplement: Supplementary file 1 — Supplementary Figures. [file 41598_2022_22284_MOESM1_ESM.pdf]
