# Supplementary material for: Morphological diversity and molecular phylogeny of five Paramecium bursaria (Alveolata, Ciliophora, Oligohymenophorea) syngens and the identification of their green algal endosymbionts
Source: Sci Rep. 2022 Oct 27;12:18089. doi: 10.1038/s41598-022-22284-z (PMC9613978; doi:10.1038/s41598-022-22284-z)
Supplement: Supplementary file 2 — Supplementary Table 1. [file 41598_2022_22284_MOESM2_ESM.pdf]

Supplementary Table S1. (**A.**) List of GenBank entries (SSU) found with the BLASTn search algorithm (100% identity) sorted according to the syngens of *Paramecium bursaria* species complex. The origin of the strains is color-coded as shown in Figure 4. The hashtag marks different assignments to syngens. Only the color-coded entries were used for the analyses presented in Figure 4. Duplicates highlighted in white were not taken into account for these analyses.

References:

Gong, J., Qing, Y., Guo, X., Warren, A. (2014). "*Candidatus Sonnebornia yantaiensis*", a member of candidate division OD1, as intracellular bacteria of the ciliated protist *Paramecium bursaria* (Ciliophora, Oligohymenophorea). Syst. Appl. Microbiol. 37, 35-41.

Hoshina, R., Kato, Y., Kamako, S., Imamura, N. (2005). Genetic evidence of "American" and "European" type symbiotic algae of *Paramecium bursaria* Ehrenberg. Plant Biol. 7, 526-532.

Hoshina, R., Hayashi, S., Imamura, N. (2006). Intraspecific genetic divergence of *Paramecium bursaria* and Reconstruction of the paramecian phylogenetic tree. Acta Protozool. 45, 377-386.

Hoshina, R., Fujiwara, Y. (2012). Photobiont flexibility in *Paramecium bursaria*: Double and triple photobiont co-habitation. Adv. Microbiol. 2, 227-233.

Lanzoni, O., Sabaneyeva, E., Modeo, L., Castelli, M., Lebedeva, N., Verni, F., Schrallhammer, M., Potekhin, A., Petroni, G. (2019). Diversity and environmental distribution of the cosmopolitan endosymbiont "*Candidatus Megaira*". Sci. Rep. 9, 1179.

Rossi, A., Boscaro, V., Carducci, D., Serra, V., Modeo, L., Verni, F., Fokin, S.I., Petroni, G. (2016). Ciliate communities and hidden biodiversity in freshwater biotopes of the Pistoia province (Tuscany, Italy). Eur. J. Protistol. 53, 11-19.

Spanner, C., Darienko, T., Biehler, T., Sonntag, B., Pröschold, T. (2020). Endosymbiotic green algae in *Paramecium bursaria*: A new isolation method and a simple diagnostic PCR approach for the identification. Diversity 12, 240.

Strüder-Kypke, M.C., Wright, A.-D.G., Fokin, S.I., Lynn, D.H. (2000). Phylogenetic relationships of the genus *Paramecium* inferred from small subunit rRNA gene sequences. Mol. Phylogenet. Evol. 14, 122–130.

Sun, M., Li, Y., Cai, X., Lui, Y., Chen, Y., Pan, X. (2021) Further insights into the phylogeny of peniculid ciliates (Ciliophora, Oligohymenophorea) based on multigene data. Mol. Phylogenet. Evol. 154, 107003.

Zhao, Y., Gentekaki, E., Yi, Z., Lin, X. (2013). Genetic differentiation of the mitochondrial cytochrome oxidase c subunit I gene in genus *Paramecium* (Protista, Ciliophora). PlosOne 8, e77044.

accession #	strain	origin	continent	reference
SYNGEN R1				
MT231330	PB-19	Poland	Europe	Spanner et al. (2020)
MT231331	CCAP 1660/11	England	Europe	Spanner et al. (2020)
MT231332	CCAP 1660/12	England	Europe	Spanner et al. (2020)
MT231333	SAG 27.96	Germany	Europe	Spanner et al. (2020)
OK318475	CCAP 1660/46	Germany	Europe	this study
OK318476	CCAP 1660/47	Germany	Europe	this study
OK318477	CCAP 1660/37	Germany	Europe	this study
OK318478	CCAP 1660/38	Germany	Europe	this study
OK318479	PB-25	Austria	Europe	this study
OK318480	CCAP 1660/1B	England	Europe	this study
OK318481	CCAP 1660/13	England	Europe	this study
AB252001	CCAP 1660/12	England	Europe	Hoshina et al. (2006)
AB252002	CCAP 1660/13	England	Europe	Hoshina et al. (2006)
SYNGEN R2				
MT231334	CCAP 1660/39	Germany	Europe	Spanner et al. (2020)
MT231335	CCAP 1660/16	Scotland	Europe	Spanner et al. (2020)
MT231336	CCAP 1660/18	Scotland	Europe	Spanner et al. (2020)
MT231337	CCAP 1660/20	Scotland	Europe	Spanner et al. (2020)
OK318482	PB-27	Austria	Europe	this study
OK318483	PB-28	Austria	Europe	this study
OK318484	CCAP 1660/17	Scotland	Europe	this study
OK318485	CCAP 1660/19	Scotland	Europe	this study
OK318486	CCAP 1660/34	Switzerland	Europe	this study
OK318487	CCAP 1660/36	Austria	Europe	this study
OK318488	PB-26	England	Europe	this study
OK318489	CCAP 1660/24	Scotland	Europe	this study
MG589318	VL12-10	Russia	Europe	Lanzoni et al. (2019)
MG589317	VL3-1	Russia	Europe	Lanzoni et al. (2019)
MG589316	1M-2	Russia	Europe	Lanzoni et al. (2019)
LN869940	aLdG3	Italy	Europe	Rossi et al. (2016)
LN869998	bLaN3	Italy	Europe	Rossi et al. (2016)
LN869938	aLdG2	Italy	Europe	Rossi et al. (2016)
LN869937	aLdG1	Italy	Europe	Rossi et al. (2016)
LN869994	bLaN1	Italy	Europe	Rossi et al. (2016)
LN870006	bLdG2	Italy	Europe	Rossi et al. (2016)
SYNGEN R3				
MT231338	PB-2	USA, MA	North America	Spanner et al. (2020)
MT231339	CCAP 1660/28	Austria	Europe	Spanner et al. (2020)
MT231340	CCAP 1660/29	Austria	Europe	Spanner et al. (2020)
MT231341	CCAP 1660/21	Chile	South America	Spanner et al. (2020)
MT231342	CCAP 1660/22	Chile	South America	Spanner et al. (2020)
MT231343	CCAP 1660/23	Chile	South America	Spanner et al. (2020)
MT231344	CCAP 1660/31	Russia	Asia	Spanner et al. (2020)
MT231345	CCAP 1660/32	Russia	Asia	Spanner et al. (2020)

accession #	strain	origin	continent	reference
OK318496	PB-11	Russia	Europe	this study
OK318497	PB-13	Italy	Europe	this study
OK318490	PB-17	Japan	Asia	this study
OK318491	CCAP 1660/26	Japan	Asia	this study
OK318492	PB-15	Japan	Asia	this study
OK318493	CCAP 1660/35	Japan	Asia	this study
OK318494	PB-16	Japan	Asia	this study
OK318495	CCAP 1660/27	Japan	Asia	this study
KF017427	Qingdao	China	Asia	Zhao et al. (2013)
AB206537	OK1	Japan	Asia	Hoshina et al. (2005)
KC495066	YD-2010	China	Asia	Gong et al. (2014)
MW031791	SM2018091001	China	Asia	Sun et al. (2021)
KC495068	YD-2010-5	China	Asia	Gong et al. (2014)
KC495070	YD-2010-7	China	Asia	Gong et al. (2014)
KC495065	YD-2010-10	China	Asia	Gong et al. (2014)
KC495064	YD-2010-1	China	Asia	Gong et al. (2014)
KC495069	YD-2010-6	China	Asia	Gong et al. (2014)
KC495067	YD-2010-4	China	Asia	Gong et al. (2014)
AB699097	AG-35	Japan	Asia	Hoshina et al. (2012)
LN870042	bOmb1	Italy	Europe	Rossi et al. (2016)
LN870052	bOmb2	Italy	Europe	Rossi et al. (2016)
MN264568	YAD1g	Japan	Asia	Tarcz et al. (unpublished)
MN264567	PrK157-2	Russia	Asia	Tarcz et al. (unpublished)
MN264566	IP	Italy	Europe	Tarcz et al. (unpublished)
AB219526	MRBG1	Australia	Australia	Hoshina et al. (2005)
AB206543	Cs2	China	Asia	Hoshina et al. (2005)
AB206542	Bnd1	Japan	Asia	Hoshina et al. (2005)
AB206541	Dd1	Japan	Asia	Hoshina et al. (2005)
AB206540	KM2	Japan	Asia	Hoshina et al. (2005)
AB206539	F36	Japan	Asia	Hoshina et al. (2005)
AB206538	So13	Japan	Asia	Hoshina et al. (2005)
AB699099	DC-3	USA, NY	North America	Hoshina et al. (2012)
AB699098	BP-11	USA, MD	North America	Hoshina et al. (2012)
SYNGEN R4				
MT231346	PB-1	USA, MA	North America	Spanner et al. (2020)
MT231347	CCAP 1660/25	USA, NC	North America	Spanner et al. (2020)
OK318498	CCAP 1660/33	Chile	South America	this study
AB252000	CCAP 1660/10	England	Europe	Hoshina et al. (2006)
AF100314	Ontario	Canada, ON	North America	Strüder-Kypke et al. (2000)
AB699100	OLG-3	USA, FL	North America	Hoshina et al. (2012)
SYNGEN R5				
MT231348	CCAP 1660/30	Russia	Europe	Spanner et al. (2020)
AB206544 #	PB-SW1	Germany	Europe	Hoshina et al. (2005)
AB206545 #	CCAP 1660/11	England	Europe	Hoshina et al. (2005)

(**B.**) List of GenBank entries (ITS) found with the BLASTn search algorithm (98-100% identity) sorted according to the syngens of *Paramecium bursaria* species complex. The origin of the strains is color-coded as shown in Figure 4. Duplicated sequences submitted to GenBank are highlighted with an asterisk. The hashtag marks different assignments to syngens. Only the color-coded entries were used for the analyses presented in Figure 4. Duplicates highlighted in white and were not taken into account for these analyses.

References:

Greczek-Stachura, M., Tarcz, S., Przybos, E. (2010). Intra-specific differentiation of *Paramecium bursaria* strains by molecular methods – Preliminary studies. Folia Biol. (Kraków) 58, 35-45.

Greczek-Stachura, M., Potekhin, A., Przybos, E., Rautian, M., Skobio, I., Tarcz, S. (2012). Identification of *Paramecium bursaria* syngens through molecular markers – comparative analysis of three loci in the nuclear and mitochondrial DNA. Protist 163, 671-685.

Hoshina, R., Hayashi, S., Imamura, N. (2006). Intraspecific genetic divergence of *Paramecium bursaria* and Reconstruction of the paramecian phylogenetic tree. Acta Protozool. 45, 377-386.

Przybos, E., Tarcz, S., Potekhin, A., Rautian, M., Prajer, M. (2010). A two-locus molecular characterization of *Paramecium calkinsi*. Protist 163, 263-273.

Spanner, C., Darienko, T., Biehler, T., Sonntag, B., Pröschold, T. (2020). Endosymbiotic green algae in *Paramecium bursaria*: A new isolation method and a simple diagnostic PCR approach for the identification. Diversity 12, 240.

accession #	variant	strain	origin	continent	reference
SYNGEN R1					
MT231333	R1a	SAG 27.96	Germany	Europe	Spanner et al. (2020)
MT231330	R1a	PB-19	Poland	Europe	Spanner et al. (2020)
MT231331	R1a	CCAP 1660/11	England	Europe	Spanner et al. (2020)
MT231332	R1a	CCAP 1660/12	England	Europe	Spanner et al. (2020)
OK318475	R1a	CCAP 1660/46	Germany	Europe	this study
OK318476	R1a	CCAP 1660/47	Germany	Europe	this study
OK318479	R1a	PB-25	Austria	Europe	this study
OK318480	R1a	CCAP 1660/1B	England	Europe	this study
OK318481	R1a	CCAP 1660/13	England	Europe	this study
OK318477	R1a	CCAP 1660/37	Germany	Europe	this study
OK318478	R1a	CCAP 1660/38	Germany	Europe	this study
JF708922	R1a	PB3	Poland	Europe	Greczek-Stachura et al. (2012)
JF708921	R1a	PB2	Poland	Europe	Greczek-Stachura et al. (2012)
JF708920	R1a*	PB1	Poland	Europe	Greczek-Stachura et al. (2012)
JF708919	R1a*	GG	Germany	Europe	Greczek-Stachura et al. (2012)
AB252038	R1a*	CCAP 1660/13	England	Europe	Hoshina et al. (2006)
AB252037	R1a*	CCAP 1660/13	England	Europe	Hoshina et al. (2006)
AB252036	R1a*	CCAP 1660/13	England	Europe	Hoshina et al. (2006)
AB252035	R1a*	CCAP 1660/13	England	Europe	Hoshina et al. (2006)
AB252031	R1a*	CCAP 1660/13	England	Europe	Hoshina et al. (2006)
AB252030	R1a*	CCAP 1660/13	England	Europe	Hoshina et al. (2006)
AB252029	R1a*	CCAP 1660/13	England	Europe	Hoshina et al. (2006)
AB252027	R1a*	CCAP 1660/13	England	Europe	Hoshina et al. (2006)
AB252026	R1a*	CCAP 1660/13	England	Europe	Hoshina et al. (2006)
AB252022	R1a*	CCAP 1660/12	England	Europe	Hoshina et al. (2006)
AB252021	R1a*	CCAP 1660/12	England	Europe	Hoshina et al. (2006)
AB252020	R1a*	CCAP 1660/12	England	Europe	Hoshina et al. (2006)
AB252019	R1a*	CCAP 1660/12	England	Europe	Hoshina et al. (2006)
AB252018	R1a*	CCAP 1660/12	England	Europe	Hoshina et al. (2006)
AB252017	R1a*	CCAP 1660/11	England	Europe	Hoshina et al. (2006)
GQ869832	R1a*	GG	Germany	Europe	Greczek-Stachura et al. (2010)
GQ869830	R1a*	PB3	Poland	Europe	Greczek-Stachura et al. (2010)
GQ869829	R1a*	PB2	Poland	Europe	Greczek-Stachura et al. (2010)
GQ869828	R1a*	PB1	Poland	Europe	Greczek-Stachura et al. (2010)
AB252015	R1b #	PB-SW1	Germany	Europe	Hoshina et al. (2006)
AB252032	R1b*	CCAP 1660/13	England	Europe	Hoshina et al. (2006)
AB252034	R1e*	CCAP 1660/13	England	Europe	Hoshina et al. (2006)
AB252033	R1c*	CCAP 1660/13	England	Europe	Hoshina et al. (2006)
AB252028	R1d*	CCAP 1660/13	England	Europe	Hoshina et al. (2006)
AB252025	R1f*	CCAP 1660/13	England	Europe	Hoshina et al. (2006)
AB252024	R1g*	CCAP 1660/12	England	Europe	Hoshina et al. (2006)
SYNGEN R2					
MT231334	R2a	CCAP 1660/39	Germany	Europe	Spanner et al. (2020)
MT231335	R2a	CCAP 1660/16	Scotland	Europe	Spanner et al. (2020)
MT231336	R2a	CCAP 1660/18	Scotland	Europe	Spanner et al. (2020)
MT231337	R2a	CCAP 1660/20	Scotland	Europe	Spanner et al. (2020)
OK318486	R2a	CCAP 1660/34	Switzerland	Europe	this study
OK318487	R2a	CCAP 1660/36	Austria	Europe	this study
OK318482	R2a	PB-27	Austria	Europe	this study
OK318483	R2a	PB-28	Austria	Europe	this study
JF708940	R2a	Hg24g	Australia	Australia	Greczek-Stachura et al. (2012)
JF708939	R2a	Hg5g	Australia	Australia	Greczek-Stachura et al. (2012)
JF708938	R2a	Bob1	Russia	Europe	Greczek-Stachura et al. (2012)
JF708937	R2a	Obv	Russia	Europe	Greczek-Stachura et al. (2012)

accession #	variant	strain	origin	continent	reference
JF708936	R2a	Ek	Russia	Europe	Greczek-Stachura et al. (2012)
JF708929	R2a	AZ17-5	Russia	Europe	Greczek-Stachura et al. (2012)
JF708924	R2a	UK	Ukraine	Europe	Greczek-Stachura et al. (2012)
JF708923	R2a	PKO	Poland	Europe	Greczek-Stachura et al. (2012)
GQ869827	R2a*	UK	Ukraine	Europe	Greczek-Stachura et al. (2010)
OK318488	R2b	PB-26	England	Europe	this study
SYNGEN R3					
MT231338	R3a	PB-2	USA, MA	North America	Spanner et al. (2020)
MT231341	R3a	CCAP 1660/21	Chile	South America	Spanner et al. (2020)
MT231342	R3a	CCAP 1660/22	Chile	South America	Spanner et al. (2020)
MT231343	R3a	CCAP 1660/23	Chile	South America	Spanner et al. (2020)
OK318495	R3a	CCAP 1660/27	Japan	Asia	this study
OK318491	R3a	CCAP 1660/26	Japan	Asia	this study
OK318494	R3a	PB-16	Japan	Asia	this study
JF708927	R3a	YAD-1g	Japan	Asia	Greczek-Stachura et al. (2012)
JF708926	R3a	SKS-4-5	Japan	Asia	Greczek-Stachura et al. (2012)
JF708925	R3a	T316	Japan	Asia	Greczek-Stachura et al. (2012)
AB252014	R3a	MRBG1	Australia	Australia	Hoshina et al. (2006)
AB252011	R3a	So13	Japan	Asia	Hoshina et al. (2006)
AB252010	R3a	OK1	Japan	Asia	Hoshina et al. (2006)
GQ869826	R3a	JT	Japan	Asia	Greczek-Stachura et al. (2010)
MT231339	R3b	CCAP 1660/28	Austria	Europe	Spanner et al. (2020)
MT231340	R3b	CCAP 1660/29	Austria	Europe	Spanner et al. (2020)
OK318493	R3b	CCAP 1660/35	Japan	Asia	this study
JF708918	R3b*	AW	Austria	Europe	Greczek-Stachura et al. (2012)
JF708917	R3b*	APS	Austria	Europe	Greczek-Stachura et al. (2012)
AB252012	R3b	F36	Japan	Asia	Hoshina et al. (2006)
GQ869834	R3b*	APS	Austria	Europe	Greczek-Stachura et al. (2012)
GQ869833	R3b*	AW	Austria	Europe	Greczek-Stachura et al. (2012)
MT231344	R3c	CCAP 1660/31	Russia	Asia	Spanner et al. (2020)
MT231345	R3c	CCAP 1660/32	Russia	Asia	Spanner et al. (2020)
JF708935	R3c*	PrK157-2	Russia	Asia	Greczek-Stachura et al. (2012)
JF708934	R3c	HZ126-6	Russia	Asia	Greczek-Stachura et al. (2012)
JF708931	R3c	HKV19-12	Russia	Asia	Greczek-Stachura et al. (2012)
JF708928	R3c*	HA45-3	Russia	Asia	Greczek-Stachura et al. (2012)
AB252013	R3c	Cs2	China	Asia	Hoshina et al. (2006)
OK318497	R3d	PB-13	Russia	Europe	this study
OK318496	R3d	PB-11	Italy	Europe	this study
GQ869825	R3d*	IP	Italy	Europe	Greczek-Stachura et al. (2012)
JF304169	R3d*	IP	Italy	Europe	Przybos et al. (2012)
JF708933	R3e	HZ75-5	Russia	Asia	Przybos et al. (2012)
JF708932	R3f	Hb51-1	Russia	Asia	Greczek-Stachura et al. (2012)
SYNGEN R4					
MT231346	R4a	PB-1	USA, MA	North America	Spanner et al. (2020)
JF708941	R4a*	AB2-32	USA, MA	North America	Greczek-Stachura et al. (2012)
AB252016	R4a	CCAP 1660/10	England	Europe	Hoshina et al. (2006)
MT231347	R4b	CCAP 1660/25	USA, NC	North America	Spanner et al. (2020)
OK318498	R4c	CCAP 1660/33	Chile	South America	this study
SYNGEN R5					
MT231348	R5	CCAP 1660/30	Russia	Europe	Spanner et al. (2020)
JF708930	R5*	AZ20-1	Russia	Europe	Greczek-Stachura et al. (2012)

(**C.**) List of GenBank entries (COI) found with the BLASTn search algorithm (98-100% identity) sorted according to the syngens of *Paramecium bursaria* species complex. The origin of the strains is color-coded as shown in Figure 4.

References:

Barth, D., Berendonk, T.U. (2011). The mitochondrial genome sequence of the ciliate *Paramecium caudatum* reveals a shift in nucleotide composition and codon usage within the genus *Paramecium*. BMC Genomics 12, 272.

Greczek-Stachura, M., Potekhin, A., Przybos, E., Rautian, M., Skobio, I., Tarcz, S. (2012). Identification of *Paramecium bursaria* syngens through molecular markers – comparative analysis of three loci in the nuclear and mitochondrial DNA. Protist 163, 671-685.

Greczek-Stachura, M., Rautian, M., Tarcz, S. (2021). *Paramecium bursaria* - A complex of five cryptic species: Mitochondrial DNA COI haplotype variation and biogeographic distribution. Diversity 13, 589.

Potekhin, A., Mayen-Estrada, R. (2020). *Paramecium* diversity and a new member of the *Paramecium aurelia* species complex described from Mexico. Diversity 12, 197.

Strueder-Kypke, M.C., Lynn, D.H. (2010). Comparative analysis of the mitochondrial cytochrome c oxidase subunit I (COI) gene in ciliates (Alveolata, Ciliophora) and evaluation of its suitability as a biodiversity marker. System. Biodivers. 8, 131-148.

Zagata, P., Greczek-Stachura, M., Tarcz, S., Rautian, M. (2015). Molecular identification of *Paramecium bursaria* syngens and studies on geographic distribution using mitochondrial cytochrome C oxidase subunit I (COI). Folia Biologica (Krakow) 63, 77-83.

Zhao, Y., Gentekaki, E., Yi, Z., Lin, X. (2013). Genetic differentiation of the mitochondrial cytochrome oxidase c subunit I gene in genus *Paramecium* (Protista, Ciliophora). PlosOne 8, e77044.

accession #	variant	strain	origin	continent	reference
SYNGEN R1					
OK356526	R1-1	87 MS-1	Russia	Europe	Greczek-Stachura et al. (2021)
JF708896	R1-1	PB2	Poland	Europe	Greczek-Stachura et al. (2012)
JF708897	R1-1	PB3	Poland	Europe	Greczek-Stachura et al. (2012)
JF708899	R1-1	UK	Ukraine	Europe	Greczek-Stachura et al. (2012)
JF708895	R1-1	PB1	Poland	Europe	Greczek-Stachura et al. (2012)
OK356523	R1-1	88 T1-3	Tajikistan	Asia	Greczek-Stachura et al. (2021)
OK356524	R1-1	UV 2-2	Ukraine	Europe	Greczek-Stachura et al. (2021)
KJ701557	R1-1	AZ 12-9	Russia	Europe	Zagata et al. (2015)
OK356527	R1-1	ShT 56	Tajikistan	Asia	Greczek-Stachura et al. (2021)
OK356529	R1-1	TR54-1	Russia	Europe	Greczek-Stachura et al. (2021)
FJ905152	R1-1	OLI-01	Canada	North America	Strueder-Kypke & Lynn (2010)
OK356531	R1-1	RN88-14	Russia	Europe	Greczek-Stachura et al. (2021)
KJ701556	R1-2	BOB130-6	Russia	Asia	Zagata et al. (2015)
JF708893	R1-3	GG = SAG 27.96	Germany	Europe	Greczek-Stachura et al. (2012)
OK356530	R1-3	Wien 4a-3	Austria	Europe	Greczek-Stachura et al. (2021)
OK356525	R1-4	RV 82-3	Russia	Europe	Greczek-Stachura et al. (2021)
KJ701558	R1-5	AS 62-9	Armenia	Asia	Zagata et al. (2015)
KJ701559	R1-6	T 24-5	Tajikistan	Asia	Zagata et al. (2015)
OK356528	R1-7	NB 2-10	Ukraine	Europe	Greczek-Stachura et al. (2021)
SYNGEN R2					
JF708911	R2-1	Ek	Russia	Europe	Greczek-Stachura et al. (2012)
JF708904	R2-1	AZ17-5	Russia	Europe	Greczek-Stachura et al. (2012)
JF708912	R2-1	Obv	Russia	Europe	Greczek-Stachura et al. (2012)
JF708913	R2-1	Bob1	Russia	Europe	Greczek-Stachura et al. (2012)
OK356532	R2-1	BL16-12	Russia	Asia	Greczek-Stachura et al. (2021)
OK356533	R2-1	BBR49-8	Russia	Asia	Greczek-Stachura et al. (2021)
KJ701560	R2-1	BBR51-1	Russia	Asia	Zagata et al. (2015)
KJ701561	R2-1	BBR174-7	Russia	Asia	Zagata et al. (2015)
OK356534	R2-1	BBR178-9	Russia	Asia	Greczek-Stachura et al. (2021)
OK356535	R2-1	BBR180-10	Russia	Asia	Greczek-Stachura et al. (2021)
OK356536	R2-1	TRB101-1	Russia	Asia	Greczek-Stachura et al. (2021)
KJ701562	R2-1	NRB217-1	Russia	Asia	Zagata et al. (2015)
KJ701563	R2-1	BBK197-2-2	Russia	Asia	Zagata et al. (2015)
OK356538	R2-1	NRB215-1	Russia	Asia	Greczek-Stachura et al. (2021)
OK356539	R2-1	MRT1-1	Russia	Asia	Greczek-Stachura et al. (2021)
KJ701564	R2-1	RA 2-1	Russia	Asia	Zagata et al. (2015)
OK356540	R2-1	02 SK-4	Russia	Europe	Greczek-Stachura et al. (2021)
OK356541	R2-1	AZ 8-2	Russia	Europe	Greczek-Stachura et al. (2021)
KJ701566	R2-1	96 Bi-2	Russia	Europe	Zagata et al. (2015)
KJ701567	R2-1	V6-1	Russia	Europe	Zagata et al. (2015)
KJ701568	R2-1	AZ 21-3	Russia	Europe	Zagata et al. (2015)
KJ701569	R2-1	AZ 20-4	Russia	Europe	Zagata et al. (2015)
KJ701570	R2-1	KT 1-1	Russia	Asia	Zagata et al. (2015)
OK356542	R2-1	BYa 129-5	Russia	Europe	Greczek-Stachura et al. (2021)
OK356543	R2-1	Mo 3-1	Russia	Europe	Greczek-Stachura et al. (2021)
OK356545	R2-1	AZ7-13	Russia	Europe	Greczek-Stachura et al. (2021)
OK356546	R2-1	AZ7-14	Russia	Europe	Greczek-Stachura et al. (2021)
OK356547	R2-1	KZH-29	Russia	Europe	Greczek-Stachura et al. (2021)
JF708898	R2-2	PKO	Poland	Europe	Greczek-Stachura et al. (2012)
JF708914	R2-3	Hg5g	Australia	Australia	Greczek-Stachura et al. (2012)
JF708915	R2-3	Hg24g	Australia	Australia	Greczek-Stachura et al. (2012)
KJ701565	R2-3	KZ-126	Russia	Europe	Zagata et al. (2015)
OK356549	R2-3	SRB9-1	Serbia	Europe	Greczek-Stachura et al. (2021)
FN421334	R2-3	D-Cran	Germany	Europe	Barth & Berendonk (2011)

accession #	variant	strain	origin	continent	reference
OK356537	R2-4	BBK196-2	Russia	Asia	Greczek-Stachura et al. (2021)
OK356544	R2-5	TR54-4	Russia	Europe	Greczek-Stachura et al. (2021)
OK356548	R2-6	ABT1-4	Russia	Asia	Greczek-Stachura et al. (2021)
SYNGEN R3					
JF708900	R3-1	T316	Japan	Asia	Greczek-Stachura et al. (2012)
JF708891	R3-1	APS	Austria	Europe	Greczek-Stachura et al. (2012)
JF708892	R3-1	AW	Austria	Europe	Greczek-Stachura et al. (2012)
JF708894	R3-1	IP	Italy	Europe	Greczek-Stachura et al. (2012)
JF708901	R3-1	SKS4-5	Japan	Asia	Greczek-Stachura et al. (2012)
OK356550	R3-1	JR-16	Japan	Asia	Greczek-Stachura et al. (2021)
OK356551	R3-1	AB 6-65	USA	North America	Greczek-Stachura et al. (2021)
KJ701572	R3-1	Cs2	China	Asia	Zagata et al. (2015)
JX082013	R3-1	Pb1C12	China	Asia	Zhao et al. (2013)
JX082014	R3-1	Pb1C13	China	Asia	Zhao et al. (2013)
JX082015	R3-1	Pb1C14	China	Asia	Zhao et al. (2013)
JX082016	R3-1	Pb1C15	China	Asia	Zhao et al. (2013)
JX082017	R3-1	Pb1C16	China	Asia	Zhao et al. (2013)
JX082018	R3-1	Pb1C17	China	Asia	Zhao et al. (2013)
JX082020	R3-1	Pb1C19	China	Asia	Zhao et al. (2013)
JX082021	R3-1	Pb1C10	China	Asia	Zhao et al. (2013)
JX082022	R3-1	Pb1C21	China	Asia	Zhao et al. (2013)
JX082023	R3-1	Pb1C22	China	Asia	Zhao et al. (2013)
JX082024	R3-1	Pb1C23	China	Asia	Zhao et al. (2013)
JX082031	R3-1	Pb1C20	China	Asia	Zhao et al. (2013)
JX082032	R3-1	Pb1C31	China	Asia	Zhao et al. (2013)
JX082033	R3-1	Pb1C32	China	Asia	Zhao et al. (2013)
JX082034	R3-1	Pb1C33	China	Asia	Zhao et al. (2013)
JX082035	R3-1	Pb1C34	China	Asia	Zhao et al. (2013)
JX082036	R3-1	Pb1C35	China	Asia	Zhao et al. (2013)
JX082037	R3-1	Pb1C36	China	Asia	Zhao et al. (2013)
JX082038	R3-1	Pb1C37	China	Asia	Zhao et al. (2013)
JX082041	R3-1	Pb1C30	China	Asia	Zhao et al. (2013)
JX082043	R3-1	Pb1C42	China	Asia	Zhao et al. (2013)
JX082044	R3-1	Pb1C43	China	Asia	Zhao et al. (2013)
JX082045	R3-1	Pb1C44	China	Asia	Zhao et al. (2013)
JX082046	R3-1	Pb1C45	China	Asia	Zhao et al. (2013)
JX082047	R3-1	Pb1C46	China	Asia	Zhao et al. (2013)
JX082049	R3-1	Pb1C48	China	Asia	Zhao et al. (2013)
JX082050	R3-1	Pb1C49	China	Asia	Zhao et al. (2013)
JX082051	R3-1	Pb1C40	China	Asia	Zhao et al. (2013)
JX082053	R3-1	Pb2C2	China	Asia	Zhao et al. (2013)
JX082054	R3-1	Pb2C3	China	Asia	Zhao et al. (2013)
JX082056	R3-1	Pb2C5	China	Asia	Zhao et al. (2013)
JX082057	R3-1	Pb3C31	China	Asia	Zhao et al. (2013)
JX082059	R3-1	Pb3C22	China	Asia	Zhao et al. (2013)
MT078144	R3-1	K11-4B	Mexico	North America	Potekhin & Mayen-Estrada (2020)
JX082039	R3-1	Pb1C38	China	Asia	Zhao et al. (2013)
JF708902	R3-2	YAD1g	Japan	Asia	Zhao et al. (2013)
JF708903	R3-2	HA45-3	Russia	Asia	Zhao et al. (2013)
JF708906	R3-2	HKV19-12	Russia	Asia	Zhao et al. (2013)
JF708907	R3-2	Hb51-1	Russia	Asia	Zhao et al. (2013)
JF708908	R3-2	HZ75-5	Russia	Asia	Zhao et al. (2013)
JF708909	R3-2	HZ126-6	Russia	Asia	Zhao et al. (2013)
JF708910	R3-2	PrK157-2	Russia	Asia	Zhao et al. (2013)
JX082060	R3-2	Pb3C12	China	Asia	Zhao et al. (2013)

accession #	variant	strain	origin	continent	reference
JX082061	R3-2	Pb3C13	China	Asia	Zhao et al. (2013)
JX082062	R3-2	Pb3C14	China	Asia	Zhao et al. (2013)
JX082063	R3-2	Pb3C11	China	Asia	Zhao et al. (2013)
JX082042	R3-3	Pb1C41	China	Asia	Zhao et al. (2013)
KJ701571	R3-4	BP-28	Russia	Asia	Zagata et al. (2015)
OK356552	R3-4	05 CB11-1	China	Asia	Greczek-Stachura et al. (2021)
JX082012	R3-5	Pb1C11	China	Asia	Zhao et al. (2013)
JX082019	R3-6	Pb1C18	China	Asia	Zhao et al. (2013)
JX082025	R3-7	Pb1C24	China	Asia	Zhao et al. (2013)
JX082026	R3-8	Pb1C25	China	Asia	Zhao et al. (2013)
JX082027	R3-9	Pb1C26	China	Asia	Zhao et al. (2013)
JX082028	R3-10	Pb1C27	China	Asia	Zhao et al. (2013)
JX082029	R3-11	Pb1C28	China	Asia	Zhao et al. (2013)
JX082030	R3-12	Pb1C29	China	Asia	Zhao et al. (2013)
JX082040	R3-13	Pb1C39	China	Asia	Zhao et al. (2013)
JX082048	R3-14	Pb1C47	China	Asia	Zhao et al. (2013)
JX082052	R3-15	Pb2C1	China	Asia	Zhao et al. (2013)
JX082055	R3-15	Pb3C4	China	Asia	Zhao et al. (2013)
JX082058	R3-16	Pb3C21	China	Asia	Zhao et al. (2013)
SYNGEN R4					
JF708916	R4	AB2-32	USA	North America	Greczek-Stachura et al. (2012)
KJ701574	R4	Ard 7	USA	North America	Zagata et al. (2015)
KJ701575	R4	Ard 9	USA	North America	Zagata et al. (2015)
OK356553	R4	Ard 10	USA	North America	Greczek-Stachura et al. (2021)
SYNGEN R5					
JF708905	R5-1	AZ20-1	Russia	Europe	Greczek-Stachura et al. (2012)
KJ701576	R5-2	BS-3	Russia	Europe	Zagata et al. (2015)
