# Supplementary material for: Morphological diversity and molecular phylogeny of five Paramecium bursaria (Alveolata, Ciliophora, Oligohymenophorea) syngens and the identification of their green algal endosymbionts
Source: Sci Rep. 2022 Oct 27;12:18089. doi: 10.1038/s41598-022-22284-z (PMC9613978; doi:10.1038/s41598-022-22284-z)
Supplement: Supplementary file 3 — Supplementary Table 2. [file 41598_2022_22284_MOESM3_ESM.pdf]

Supplementary Table 2: Morphometric data on the investigated *Paramecium bursaria* strains. **CV** = coefficient of variation in %, **DSN** = dry Silver-Nitrate Staining, **M** = median, **IV** = in vivo, **Max** = maximum, **Mean** = arithmetic mean, **Meth** = methods, **Min** = minimum, **n** = number of specimens investigated, **P** = after protargol staining, **SD** = standard deviation. The asterisk marks the investigation of two clones of the same strain to demonstrate the within-strain variability.

Character	Syngen	Strain	Meth.	Mean	M	SD	SE	CV	Min	Max	n
**Cell length [µm]**	**R1**	**SAG 27.96**	IV	106.8	107	14.4	3.1	13	70.3	128.7	21
		**PB-25**	IV	135.5	134.2	11.3	2.5	8	115.8	163.6	21
	**R2**	**CCAP 1660/36**	IV	129.7	133.6	17.3	3.8	13	92.3	167.2	21
		**CCAP 1660/34**	IV	110.3	113.1	12.7	2.8	12	81	135.5	21
	**R3**	**CCAP 1660/26**	IV	138.5	139.3	10.5	2.3	8	112.8	153.1	21
		**CCAP 1660/28**	IV	101.7	103.1	9.6	2.1	9	80.4	116.8	21
		**CCAP 1660/31**	IV	117.5	118.0	9.8	2.1	8	99.6	138	21
	**R4**	**CCAP 1660/25***	IV	107.5	102.8	26.3	5.7	24	65.3	149.5	21
		**CCAP 1660/25***	IV	116.2	113.8	19.2	4.2	17	71.5	149.1	21
		**CCAP 1660/33**	IV	145.1	146.2	20.7	4.5	14	97.4	178.7	21
	**R5**	**CCAP 1660/30**	IV	176.1	176.4	7.3	1.6	4	160.7	194.3	21
**Cell width [µm]**	**R1**	**SAG 27.96**	IV	56.8	59.8	6.7	1.5	12	44.1	64.6	21
		**PB-25**	IV	66.6	65.9	4.5	1.0	7	56	73.2	21
	**R2**	**CCAP 1660/36**	IV	56.4	57.7	9.0	2.0	16	34.7	70	21
		**CCAP 1660/34**	IV	73.4	75.7	8.4	1.8	11	48.4	83.2	21
	**R3**	**CCAP 1660/26**	IV	61.3	61.2	4.4	1.0	7	52.8	68.1	21
		**CCAP 1660/28**	IV	64.3	65.0	5.6	1.2	9	51.1	72.6	21
		**CCAP 1660/31**	IV	56.8	57.5	5.0	1.1	9	48.6	65.6	21
	**R4**	**CCAP 1660/25***	IV	54.8	54.1	10.8	2.4	20	36.6	70.6	21
		**CCAP 1660/25***	IV	58.5	58.3	6.4	1.4	11	45.6	67.9	21
		**CCAP 1660/33**	IV	67.3	68.4	9.3	2.0	14	44	79.2	21
	**R5**	**CCAP 1660/30**	IV	88.9	88.6	5.7	1.3	6	75.6	99	21
**Caudal cilia, length [µm]**	**R1**	**SAG 27.96**	IV	12.4	11.6	2.3	0.5	18	9.3	16.8	21
		**PB-25**	IV	15.8	15.8	2.0	0.4	13	11.9	18.6	21
	**R2**	**CCAP 1660/36**	IV	15.2	15.2	2.4	0.5	15	11.4	19.5	21
		**CCAP 1660/34**	IV	12.4	12.3	1.1	0.2	9	10.5	14.5	21
	**R3**	**CCAP 1660/26**	IV	16.9	16.6	1.5	0.3	9	14.3	19.3	21
		**CCAP 1660/28**	IV	10.5	10.4	1.3	0.3	13	8.4	14.4	21
		**CCAP 1660/31**	IV	14.8	15.0	1.4	0.3	10	11.9	17	21
	**R4**	**CCAP 1660/25***	IV	16.4	16.7	1.5	0.3	9	13.5	19.8	21
		**CCAP 1660/25***	IV	15.7	15.2	1.6	0.3	10	13.4	19	21
		**CCAP 1660/33**	IV	16.2	16.8	1.9	0.4	11	11.8	18.2	21
	**R5**	**CCAP 1660/30**	IV	17.8	17.1	2.7	0.6	15	13.5	24.6	21
**Macronucleus, length [µm]**	**R1**	**SAG 27.96**	IV	31.1	31.3	3.5	0.8	11	25	37.8	21
		**PB-25**	IV	30.3	30.4	2.3	0.5	8	26.9	36	21
	**R2**	**CCAP 1660/36**	IV	36.2	36.6	4.7	1.0	13	27	45.6	21
		**CCAP 1660/34**	IV	27.9	27.2	3.3	0.7	12	23.6	37.1	21
	**R3**	**CCAP 1660/26**	IV	45.2	45.5	10.7	2.3	24	35.9	52.9	21
		**CCAP 1660/28**	IV	28.2	28.2	3.9	0.9	14	21.4	37.4	21
		**CCAP 1660/31**	IV	28.1	27.3	3.1	0.7	11	22.7	34.4	21
	**R4**	**CCAP 1660/25***	IV	34.2	35.2	9.2	2.1	27	17.7	43.4	20
		**CCAP 1660/25***	IV	34.4	34.0	13.3	2.9	39	24.2	44.1	21
		**CCAP 1660/33**	IV	45.1	46.1	11.1	2.5	25	29.4	52.8	20
	**R5**	**CCAP 1660/30**	IV	36.8	38.2	5.5	1.2	15	24.1	46.6	21
**Macronucleus, width [µm]**	**R1**	**SAG 27.96**	IV	16.9	16.7	2.6	0.6	16	11.4	22.3	21
		**PB-25**	IV	16.6	16.6	2.0	0.4	12	13	21.1	21
	**R2**	**CCAP 1660/36**	IV	16.8	17.9	2.9	0.6	17	10.4	21.3	21
		**CCAP 1660/34**	IV	22.5	22.3	3.2	0.7	14	17.9	31.8	21
	**R3**	**CCAP 1660/26**	IV	16.8	16.9	4.3	0.9	25	12.2	21.7	21
		**CCAP 1660/28**	IV	25.0	24.5	3.4	0.7	13	20.5	31.4	21
		**CCAP 1660/31**	IV	15.1	15.2	1.8	0.4	12	12.7	18.7	21
	**R4**	**CCAP 1660/25***	IV	16.6	17.2	4.9	1.1	30	9.8	22	20
		**CCAP 1660/25***	IV	17.4	17.3	7.1	1.5	41	11.6	23	21
		**CCAP 1660/33**	IV	21.2	21.8	5.9	1.3	28	12.3	28.8	20
	**R5**	**CCAP 1660/30**	IV	26.8	27.2	2.5	0.5	9	19.3	31.2	21

Character	Syngen	Strain	Meth.	Mean	M	SD	SE	CV	Min	Max	n
Micronucleus, length [µm]	R1	SAG 27.96	IV	15.9	16.0	2.1	0.5	13	12.5	20	21
		PB-25	IV	13.3	12.9	1.2	0.3	9	11.4	16.4	21
	R2	CCAP 1660/36	IV	12.9	12.4	2.0	0.4	15	10.4	18.2	21
		CCAP 1660/34	IV	-	-	-	-	-	-	-	21
	R3	CCAP 1660/26	IV	12.2	12.3	1.5	0.3	12	9	15.2	21
		CCAP 1660/28	IV	-	-	-	-	-	-	-	21
		CCAP 1660/31	IV	12.8	12.1	1.9	0.4	15	10.3	16.7	21
	R4	CCAP 1660/25*	IV	14.2	14.0	3.6	0.8	26	10.9	17.7	20
		CCAP 1660/25*	IV	13.3	13.7	3.8	0.8	29	8.2	18.2	21
		CCAP 1660/33	IV	15.5	15.2	3.6	0.8	23	13.7	17.8	20
	R5	CCAP 1660/30	IV	16.0	15.8	2.0	0.4	13	12.6	19.9	21
Micronucleus, width [µm]	R1	SAG 27.96	IV	7.4	7.6	0.7	0.2	9	5.9	8.3	21
		PB-25	IV	6.6	6.8	0.6	0.1	9	4.9	7.4	21
	R2	CCAP 1660/36	IV	6.7	6.7	0.8	0.2	12	5.3	8.5	21
		CCAP 1660/34	IV	-	-	-	-	-	-	-	21
	R3	CCAP 1660/26	IV	4.8	4.8	0.4	0.1	8	4.1	5.6	21
		CCAP 1660/28	IV	-	-	-	-	-	-	-	21
		CCAP 1660/31	IV	4.0	4.1	0.4	0.1	11	3.4	4.8	21
	R4	CCAP 1660/25*	IV	6.1	6.2	1.8	0.4	30	4	9.6	20
		CCAP 1660/25*	IV	5.8	5.8	1.7	0.4	29	4	7.9	21
		CCAP 1660/33	IV	6.6	6.8	1.6	0.4	24	5.3	7.6	20
	R5	CCAP 1660/30	IV	6.4	6.3	1.3	0.3	21	3.9	9.3	21
Number of contractile vacuoles (CV)	R1	SAG 27.96	IV	2.0	2.0	0.0	0.0	0	2	2	21
		PB-25	IV	2.0	2.0	0.2	0.0	11	1	2	21
	R2	CCAP 1660/36	IV	2.0	2.0	0.6	0.1	31	1	3	21
		CCAP 1660/34	IV	2.0	2.0	0.5	0.1	21	2	3	21
	R3	CCAP 1660/26	IV	2.0	2.0	0.2	0.0	11	2	3	21
		CCAP 1660/28	IV	2.0	2.0	0.6	0.1	33	1	3	21
		CCAP 1660/31	IV	2.0	2.0	0.4	0.1	19	1	3	21
	R4	CCAP 1660/25*	IV	2.0	2.0	0.6	0.1	35	1	3	21
		CCAP 1660/25*	IV	2.0	2.0	0.6	0.1	31	1	3	21
		CCAP 1660/33	IV	2.0	2.0	0.2	0.0	11	1	2	21
	R5	CCAP 1660/30	IV	2.0	2.0	0.7	0.2	44	1	3	21
Number of excretory pores in anterior CV	R1	SAG 27.96	IV	1.7	2.0	0.5	0.1	29	1	2	21
		PB-25	IV	2.1	2.0	0.3	0.1	14	2	3	21
	R2	CCAP 1660/36	IV	2.1	2.0	0.8	0.2	39	1	3	21
		CCAP 1660/34	IV	1.5	1.5	0.6	0.1	40	1	2	21
	R3	CCAP 1660/26	IV	2.3	2.0	0.5	0.1	21	2	3	21
		CCAP 1660/28	IV	1.0	1.0	0.5	0.1	46	1	1	21
		CCAP 1660/31	IV	1.6	2.0	0.6	0.1	36	1	3	21
	R4	CCAP 1660/25*	IV	1.9	2.0	1.0	0.2	53	1	3	21
		CCAP 1660/25*	IV	1.5	1.5	0.9	0.2	57	1	2	21
		CCAP 1660/33	IV	2.0	2.0	0.0	0.0	0	2	2	21
	R5	CCAP 1660/30	IV	1.9	2.0	1.1	0.2	57	1	4	21
Number of excretory pores in median CV	R1	SAG 27.96	IV	-	-	-	-	-	0	0	21
		PB-25	IV	-	-	-	-	-	0	0	21
	R2	CCAP 1660/36	IV	1.7	2.0	0.6	0.1	37	1	2	21
		CCAP 1660/34	IV	1.3	1.0	0.7	0.2	57	1	2	21
	R3	CCAP 1660/26	IV	1.5	1.5	0.5	0.1	32	1	2	21
		CCAP 1660/28	IV	1.3	1.0	0.5	0.1	43	1	2	21
		CCAP 1660/31	IV	1.5	1.5	0.5	0.1	32	1	2	21
	R4	CCAP 1660/25*	IV	1.5	1.5	0.6	0.1	43	1	2	21
		CCAP 1660/25*	IV	1.3	1.0	0.7	0.2	56	1	2	21
		CCAP 1660/33	IV	-	-	-	-	-	0	0	21
	R5	CCAP 1660/30	IV	1.7	2.0	0.9	0.2	51	1	2	21

Character	Syngen	Strain	Meth.	Mean	M	SD	SE	CV	Min	Max	n
**Number of excretory pores in posterior CV**	**R1**	**SAG 27.96**	IV	1.9	2.0	0.3	0.1	16	1	2	21
		**PB-25**	IV	2.0	2.0	0.5	0.1	27	1	3	21
	**R2**	**CCAP 1660/36**	IV	1.9	2.0	0.9	0.2	46	1	3	21
		**CCAP 1660/34**	IV	1.4	1.0	0.6	0.1	45	1	2	21
	**R3**	**CCAP 1660/26**	IV	2.0	2.0	0.7	0.2	37	1	3	21
		**CCAP 1660/28**	IV	1.2	1.0	0.7	0.1	57	1	3	21
		**CCAP 1660/31**	IV	1.3	1.0	0.5	0.1	41	1	2	21
	**R4**	**CCAP 1660/25***	IV	1.6	2.0	0.9	0.2	52	1	3	21
		**CCAP 1660/25***	IV	1.6	2.0	0.7	0.2	47	1	2	21
		**CCAP 1660/33**	IV	1.9	2.0	0.5	0.1	27	1	2	21
	**R5**	**CCAP 1660/30**	IV	1.8	2.0	1.0	0.2	57	1	3	21
**Length of larger symbiotic algae [µm]**	**R1**	**SAG 27.96**	IV	4.9	5.0	0.4	0.1	7	4.3	5.8	21
		**PB-25**	IV	6.1	6.0	0.6	0.1	9	5.2	7.4	21
	**R2**	**CCAP 1660/36**	IV	5.9	6.1	0.5	0.1	9	4.7	6.9	21
		**CCAP 1660/34**	IV	5.0	5.0	0.3	0.1	5	4.5	5.6	21
	**R3**	**CCAP 1660/26**	IV	5.8	5.7	0.5	0.1	9	4.7	7.1	21
		**CCAP 1660/28**	IV	5.0	5.0	0.4	0.1	8	4.4	5.6	21
		**CCAP 1660/31**	IV	4.9	4.9	0.4	0.1	8	4.2	5.6	21
	**R4**	**CCAP 1660/25***	IV	5.4	5.5	0.5	0.1	9	4.6	6.2	21
		**CCAP 1660/25***	IV	5.3	5.2	0.3	0.1	6	4.8	6.1	21
		**CCAP 1660/33**	IV	3.7	3.8	0.3	0.1	9	2.8	4.2	21
	**R5**	**CCAP 1660/30**	IV	5.5	5.5	0.3	0.1	6	4.9	6.3	21
**Width of larger symbiotic algae [µm]**	**R1**	**SAG 27.96**	IV	4.6	4.7	0.3	0.1	7	3.9	5.1	21
		**PB-25**	IV	5.6	5.5	0.5	0.1	8	5.2	6.9	21
	**R2**	**CCAP 1660/36**	IV	5.5	5.5	0.5	0.1	9	4.5	6.4	21
		**CCAP 1660/34**	IV	4.5	4.6	0.3	0.1	8	3.8	5.2	21
	**R3**	**CCAP 1660/26**	IV	5.2	5.2	0.5	0.1	10	4.1	6.4	21
		**CCAP 1660/28**	IV	4.3	4.2	0.6	0.1	15	3.1	5.3	21
		**CCAP 1660/31**	IV	4.0	4.1	0.5	0.1	12	3	4.8	21
	**R4**	**CCAP 1660/25***	IV	4.9	4.8	0.4	0.1	8	4.2	5.8	21
		**CCAP 1660/25***	IV	4.8	4.9	0.4	0.1	9	3.9	5.6	21
		**CCAP 1660/33**	IV	3.3	3.5	0.3	0.1	10	2.7	3.7	21
	**R5**	**CCAP 1660/30**	IV	5.1	5.1	0.4	0.1	8	4.6	6.1	21
**Length of smaller symbiotic algae [µm]**	**R1**	**SAG 27.96**	IV	3.3	3.4	0.4	0.1	13	2.3	4	21
		**PB-25**	IV	4.4	4.3	0.4	0.1	9	3.7	5.2	21
	**R2**	**CCAP 1660/36**	IV	4.2	4.1	0.4	0.1	11	3.5	4.9	21
		**CCAP 1660/34**	IV	3.7	3.7	0.3	0.1	9	3.1	4.4	21
	**R3**	**CCAP 1660/26**	IV	4.1	4.1	0.4	0.1	10	3.4	4.8	21
		**CCAP 1660/28**	IV	3.3	3.3	0.5	0.1	16	2.6	4.4	21
		**CCAP 1660/31**	IV	3.7	3.7	0.4	0.1	10	3	4.3	21
	**R4**	**CCAP 1660/25***	IV	3.8	3.8	0.5	0.1	13	2.8	4.8	21
		**CCAP 1660/25***	IV	4.0	4.1	0.5	0.1	14	3.1	5	21
		**CCAP 1660/33**	IV	2.5	2.5	0.4	0.1	14	2	3.1	21
	**R5**	**CCAP 1660/30**	IV	4.2	4.1	0.3	0.1	8	3.8	4.8	21
**Width of smaller symbiotic algae [µm]**	**R1**	**SAG 27.96**	IV	2.4	2.4	0.4	0.1	17	1.7	3.2	21
		**PB-25**	IV	3.7	3.8	0.4	0.1	11	3	4.5	21
	**R2**	**CCAP 1660/36**	IV	3.6	3.5	0.5	0.1	14	2.7	4.6	21
		**CCAP 1660/34**	IV	3.1	3.2	0.5	0.1	15	1.8	3.8	21
	**R3**	**CCAP 1660/26**	IV	3.5	3.5	0.4	0.1	12	2.8	4.3	21
		**CCAP 1660/28**	IV	2.7	2.7	0.5	0.1	18	1.8	3.6	21
		**CCAP 1660/31**	IV	3.0	3.0	0.4	0.1	13	2.2	3.5	21
	**R4**	**CCAP 1660/25***	IV	3.3	3,4	0.4	0.1	12	2.4	3.8	21
		**CCAP 1660/25***	IV	3.2	3.2	0.3	0.1	10	2.7	3.8	21
		**CCAP 1660/33**	IV	2.1	2.2	0.4	0.1	18	1.4	2.7	21
	**R5**	**CCAP 1660/30**	IV	3.5	3.4	0.3	0.1	9	3	4.2	21

Character	Syngen	Strain	Meth.	Mean	M	SD	SE	CV	Min	Max	n
**Extrusomes, length [µm]**	**R1**	**SAG 27.96**	IV	5.0	4.8	1.3	0.3	26	3.5	6.4	20
		**PB-25**	IV	5.2	5.1	1.2	0.3	23	4.8	6.1	20
	**R2**	**CCAP 1660/36**	IV	5.1	5.1	0.3	0.1	6	4.4	5.8	21
		**CCAP 1660/34**	IV	4.6	4.5	0.3	0.1	7	4.1	5.3	21
	**R3**	**CCAP 1660/26**	IV	5.5	5.5	0.4	0.1	7	5.0	6.4	21
		**CCAP 1660/28**	IV	4.6	4.5	0.5	0.1	10	3.7	5.7	21
		**CCAP 1660/31**	IV	4.3	4.4	0.4	0.1	10	3.6	5.1	21
	**R4**	**CCAP 1660/25***	IV	4.3	4.3	0.4	0.1	9	3.6	5.5	21
		**CCAP 1660/25***	IV	4.9	4.8	0.3	0.1	7	4.4	5.5	21
		**CCAP 1660/33**	IV	5.7	5.7	0.5	0.1	9	4.9	6.6	21
	**R5**	**CCAP 1660/30**	IV	6.1	6.1	0.4	0.1	6	5.3	6.8	21
**Number of ciliary rows / 20µm**	**R1**	**SAG 27.96**	DSN	18.1	18	2.5	0.5	14	15	22	21
		**PB-25**	DSN	17.8	18	2.9	0.6	16	14	22	21
	**R2**	**CCAP 1660/36**	DSN	16.7	16	2.3	0.5	14	14	22	20
		**CCAP 1660/34**	DSN	15.1	15	1.2	0.3	8	13	17	21
	**R3**	**CCAP 1660/26**	DSN	17.3	18	2.1	0.5	12	14	20	21
		**CCAP 1660/28**	DSN	16.1	15	2.1	0.5	13	14	20	21
		**CCAP 1660/31**	DSN	15.1	15	1.7	0.4	11	12	18	21
	**R4**	**CCAP 1660/25***	DSN	15.6	15	1.3	0.3	8	14	18	21
		**CCAP 1660/25***	DSN	16.6	16	1.7	0.4	10	14	19	21
		**CCAP 1660/33**	DSN	15.7	16	1.1	0.2	7	14	18	21
	**R5**	**CCAP 1660/30**	DSN	15.8	16	1.4	0.3	9	13	19	21
