# Supplementary material for: Morphological diversity and molecular phylogeny of five Paramecium bursaria (Alveolata, Ciliophora, Oligohymenophorea) syngens and the identification of their green algal endosymbionts
Source: Sci Rep. 2022 Oct 27;12:18089. doi: 10.1038/s41598-022-22284-z (PMC9613978; doi:10.1038/s41598-022-22284-z)
Supplement: Supplementary file 4 — Supplementary Table 3. [file 41598_2022_22284_MOESM4_ESM.pdf]

Supplementary Table S3. Values for 18 principal components.

	Standard deviation	Proportion of Variance	Cumulative Proportion
PC1	2.0596	0.2357	0.2357
PC2	1.9343	0.2079	0.4435
PC3	1.6550	0.1522	0.5957
PC4	1.18668	0.07823	0.67392
PC5	1.01053	0.05673	0.73065
PC6	0.93787	0.04887	0.77952
PC7	0.80845	0.03631	0.81583
PC8	0.80711	0.03619	0.85202
PC9	0.76541	0.03255	0.88457
PC10	0.66652	0.02468	0.90925
PC11	0.61562	0.02105	0.93030
PC12	0.57806	0.01856	0.94887
PC13	0.51574	0.01478	0.96365
PC14	0.45861	0.01168	0.97533
PC15	0.45543	0.01152	0.98685
PC16	0.3202	0.0057	0.9926
PC17	0.2783	0.0043	0.9969
PC18	0.23813	0.00315	100.000
