# Supplementary material for: Morphological diversity and molecular phylogeny of five Paramecium bursaria (Alveolata, Ciliophora, Oligohymenophorea) syngens and the identification of their green algal endosymbionts
Source: Sci Rep. 2022 Oct 27;12:18089. doi: 10.1038/s41598-022-22284-z (PMC9613978; doi:10.1038/s41598-022-22284-z)
Supplement: Supplementary file 5 — Supplementary Table 4. [file 41598_2022_22284_MOESM5_ESM.pdf]

Supplementary Table S4. Partial tabulation of PCA of correlation matrix of 18 features of *Paramecium* strains (only the first five principal components are included).

	PC1	PC2	PC3	PC4	PC5
**Algal species**	**0.04015865**	**-0.107397709**	**0.506051033**	**-0.057621727**	**0.14323817**
**Number of excretory pores in anterior contractile vacuole**	**0.01431971**	**0.049013860**	**0.188299811**	**-0.601892194**	**0.29330686**
**Body cell length**	**-0.29472968**	**0.345284255**	**0.087388592**	**-0.054247243**	**0.13759686**
**Body cell width**	**-0.29616260**	**0.336330740**	**0.079258780**	**0.132558041**	**0.15171665**
**Caudal cilia, length**	**-0.17382208**	**0.231029389**	**-0.185826054**	**-0.210803945**	**-0.22500241**
**Number of ciliary rows**	**0.12592686**	**0.060295403**	**0.241621673**	**-0.031982415**	**0.06111634**
**Extrusome length**	**-0.23749045**	**0.247783472**	**0.152438206**	**-0.139588870**	**0.20819634**
**Geographic region**	**-0.23760203**	**-0.270084264**	**-0.241916726**	**-0.211090225**	**-0.25145048**
**Large symbiotic algae, length**	**0.30213406**	**0.341953211**	**0.004530424**	**-0.013964968**	**-0.18352952**
**Large symbiotic algae, width**	**0.28179392**	**0.353512942**	**0.063037455**	**0.008598303**	**-0.21099561**
**Macronucleus, length**	**-0.29658757**	**0.098500563**	**0.069798425**	**-0.424012979**	**-0.22227652**
**Macronucleus, width**	**-0.34241549**	**0.245754010**	**0.058767655**	**0.194013766**	**0.07944501**
**Micronucleus, length**	**-0.24932292**	**0.001900665**	**0.234051360**	**0.387470550**	**-0.29060372**
**Micronucleus, width**	**-0.07955452**	**0.028762776**	**0.428628822**	**0.227638131**	**-0.25217825**
**Number of excretory pores in posterior contractile vacuole**	**0.01001578**	**-0.062869777**	**0.280944904**	**-0.270698283**	**-0.61584135**
**Small symbiotic algae, length**	**0.26041094**	**0.338769095**	**-0.125383006**	**0.052376658**	**-0.05169276**
**Small symbiotic algae, width**	**0.23291900**	**0.353181299**	**-0.152676600**	**-0.071474779**	**-0.08999092**
**Syngen affiliation**	**-0.31323251**	**0.078010744**	**-0.388689925**	**0.060053716**	**-0.13628159**
